# Supplementary material for: Estimating the size of hard to sample populations: A comprehensive study on female sex workers and sexually exploited minors in Rwanda using privatized network sampling in 2023
Source: PLoS One. 2025 Aug 21;20(8):e0329772. doi: 10.1371/journal.pone.0329772 (PMC12370040; doi:10.1371/journal.pone.0329772)

PLOS ONE

Population size estimation of female sex workers and sexually exploited minors using privatized network sampling, Rwanda, 2023.

--Manuscript Draft--

**Manuscript Number:**	PONE-D-23-42748
**Article Type:**	Research Article
**Full Title:**	Population size estimation of female sex workers and sexually exploited minors using privatized network sampling, Rwanda, 2023.
**Short Title:**	Female Sex Workers Population Size Estimation: Privatized Network Sampling, Rwanda, 2023.
**Corresponding Author:**	Elysee TUYISHIME, MSc<br>University of Rwanda College of Business and Economics<br>Kigali, RWANDA
**Keywords:**	Female Sex Workers; Population Size Estimation; HIV; Privatize Network Sampling; Respondent Driven Sampling
**Abstract:**	Introduction: Female sex workers (FSW) are at increased risk of HIV and other STI. In addition, the burden of HIV infection among this group is much higher when compared to adult females in the general population. Estimating the number of FSW helps HIV/STI prevention through program design, planning, and implementation in Rwanda.<br>Methods: Data were collected from May 8th to June 24th, 2023, across 10 study sites countywide. Privatized network sampling (PNS) was used, which is a population size estimation method that uses the network information collected within a bio-behavioral survey (BBS) that used respondent-driven sampling (RDS). To estimate the FSW and sexually exploited minors' population size, three PNS estimators were used: Cross-Sample, Cross-Alter, and Cross-Network.<br>Results: The national-level FSW population size was estimated at 98,587 (95% CI: 82,978 – 114,196), corresponding to 2.3% of the total adult female population aged 15 years and above in Rwanda. We estimated that in the City of Kigali, 5.3%, in the West Province, 2.2%, in the East and South province, 1.7% each, and in the North province 1.6% were FSW as % of adult female population 15 years of age and older who were living in Kigali and these Provinces. We estimated that in the City of Kigali, 5.3%, in the West Province, 2.2%, in the East and South province, 1.7% each, and in the North province 1.6% were FSW as % of adult female population 15 years of age and older who were living in Kigali and these Provinces.<br>Conclusion: This was the first time that PNS was implemented as a PSE method in Rwanda, adding to the emerging tools that we have in the hard-to-reach PSE field. The PSE provides fundamental information to design, plan, and implement programs for FSW at the provincial level in Rwanda. Furthermore, these estimates will help to generate positive policy changes and to advocate for resources that will help in the effort to achieve a sustained HIV epidemic control in the country.
**Order of Authors:**	Elysee TUYISHIME, MSc
	Catherine Kayitesi, BSc
	Eric REMERA, PhD
	Samuel Sewava Malamba, PhD
	Ignace Habimana Kabano, PhD
	Angela Unna Chukwu, Prof
**Additional Information:**	
**Question**	**Response**
**Financial Disclosure**<br><br>Enter a financial disclosure statement that	The author(s) received no specific funding for this work.

Powered by Editorial Manager® and ProduXion Manager® from Aries Systems Corporation

describes the sources of funding for the work included in this submission. Review the submission guidelines for detailed requirements. View published research articles from *PLOS ONE* for specific examples.<br><br>This statement is required for submission and **will appear in the published article** if the submission is accepted. Please make sure it is accurate.<br><br>**Funded studies**<br>Enter a statement with the following details:<br>• Initials of the authors who received each award<br>• Grant numbers awarded to each author<br>• The full name of each funder<br>• URL of each funder website<br>• Did the sponsors or funders play any role in the study design, data collection and analysis, decision to publish, or preparation of the manuscript?<br><br>Did you receive funding for this work?	
**Competing Interests**<br><br>Use the instructions below to enter a competing interest statement for this submission. On behalf of all authors, disclose any competing interests that could be perceived to bias this work—acknowledging all financial support and any other relevant financial or non-financial competing interests.<br><br>This statement is required for submission and **will appear in the published article** if the submission is accepted. Please make sure it is accurate and that any funding sources listed in your Funding Information later in the submission form are also declared in your Financial Disclosure statement.<br><br>View published research articles from *PLOS ONE* for specific examples.	I have read the journal's policy and the authors of this manuscript have no competing interests.

**NO authors have competing interests**<br><br>Enter: *The authors have declared that no competing interests exist.*<br><br>**Authors with competing interests**<br><br>Enter competing interest details beginning with this statement:<br><br>*I have read the journal's policy and the authors of this manuscript have the following competing interests: [insert competing interests here]*<br><br>* typeset	
**Ethics Statement**<br><br>Enter an ethics statement for this submission. This statement is required if the study involved:<br><br>• Human participants<br>• Human specimens or tissue<br>• Vertebrate animals or cephalopods<br>• Vertebrate embryos or tissues<br>• Field research<br><br>Write "N/A" if the submission does not require an ethics statement.<br><br>General guidance is provided below. Consult the submission guidelines for detailed instructions. **Make sure that all information entered here is included in the Methods section of the manuscript.**	The survey was reviewed and approved by the Rwanda National Ethics Committee. Data collection staff completed training on human subjects' research and signed a confidentiality agreement before the start of enrollment. All participants provided informed consent before enrollment in the survey.

**Format for specific study types**<br><br>**Human Subject Research (involving human participants and/or tissue)**<br>• Give the name of the institutional review board or ethics committee that approved the study<br>• Include the approval number and/or a statement indicating approval of this research<br>• Indicate the form of consent obtained (written/oral) or the reason that consent was not obtained (e.g. the data were analyzed anonymously)<br><br>**Animal Research (involving vertebrate animals, embryos or tissues)**<br>• Provide the name of the Institutional Animal Care and Use Committee (IACUC) or other relevant ethics board that reviewed the study protocol, and indicate whether they approved this research or granted a formal waiver of ethical approval<br>• Include an approval number if one was obtained<br>• If the study involved *non-human primates*, add *additional details* about animal welfare and steps taken to ameliorate suffering<br>• If anesthesia, euthanasia, or any kind of animal sacrifice is part of the study, include briefly which substances and/or methods were applied<br><br>**Field Research**<br><br>Include the following details if this study involves the collection of plant, animal, or other materials from a natural setting:<br>• Field permit number<br>• Name of the institution or relevant body that granted permission	
**Data Availability**<br><br>Authors are required to make all data underlying the findings described fully available, without restriction, and from the time of publication. PLOS allows rare exceptions to address legal and ethical concerns. See the PLOS Data Policy and FAQ for detailed information.	No - some restrictions will apply

Powered by Editorial Manager® and ProduXion Manager® from Aries Systems Corporation

A Data Availability Statement describing where the data can be found is required at submission. Your answers to this question constitute the Data Availability Statement and **will be published in the article**, if accepted.<br><br>**Important:** Stating 'data available on request from the author' is not sufficient. If your data are only available upon request, select 'No' for the first question and explain your exceptional situation in the text box.<br><br>Do the authors confirm that all data underlying the findings described in their manuscript are fully available without restriction?	
**Describe where the data may be found in full sentences. If you are copying our sample text, replace any instances of XXX with the appropriate details.**<br><br>• If the data are **held or will be held in a public repository**, include URLs, accession numbers or DOIs. If this information will only be available after acceptance, indicate this by ticking the box below. For example: *All XXX files are available from the XXX database (accession number(s) XXX, XXX.*).<br>• If the data are all contained **within the manuscript and/or Supporting Information files**, enter the following: *All relevant data are within the manuscript and its Supporting Information files.*<br>• If neither of these applies but you are able to provide **details of access elsewhere**, with or without limitations, please do so. For example:<br><br>*Data cannot be shared publicly because of [XXX]. Data are available from the XXX Institutional Data Access / Ethics Committee (contact via XXX) for researchers who meet the criteria for access to confidential data.*<br><br>*The data underlying the results presented in the study are available from (include the name of the third party*	The data underlying the results presented in the study are available for researchers who meet set criteria upon request and approval by RBC (data access requests are sent at: 'info@rbc.gov.rw').

and contact information or URL).<br>• This text is appropriate if the data are owned by a third party and authors do not have permission to share the data.<br><br>* typeset	
Additional data availability information:	

Manuscript

Click here to access/download;Manuscript;121923_FSW PSE_Privatized Network Sampling_Rwanda 2023.docx

Population size estimation of female sex workers and sexually exploited minors using privatized network sampling, Rwanda, 2023.

Remove the period

Elysee Tuyishime[1], Kayitesi Catherine[2], Eric Remera[3], Samuel S. Malamba[4], Ignace Kabano[1], Angela Unna Chukwu[1,5]

Author's affiliation:

[1] University of Rwanda - African Center of Excellence in Data Science (ACE-DS), Rwanda.

[2] HIV/AIDS and STIs Division, Institute of HIV Disease Prevention and Control, Rwanda Biomedical Centre (RBC).

[3] Research, Innovation and Data Science Division, Institute of HIV Disease Prevention and Control, Rwanda Biomedical Centre (RBC).

[4] Health Research and Evaluation Consultant, Uganda.

[5] Department of Statistics, University of Ibadan/Nigeria

Abstract

Introduction: Female sex workers (FSW) are at increased risk of HIV and other STI. In addition, the burden of HIV infection among this group is much higher when compared to adult females in the general population. Estimating the number of FSW helps HIV/STI prevention through program design, planning, and implementation in Rwanda.

Methods: Data were collected from May 8th to June 24th, 2023, across 10 study sites countywide. Privatized network sampling (PNS) was used, which is a population size estimation method that uses the network information collected within a bio-behavioral survey (BBS) that used respondent-driven sampling (RDS). To estimate the FSW and sexually exploited minors' population size, three PNS estimators were used: Cross-Sample, Cross-Alter, and Cross-Network.

Results: The national-level FSW population size was estimated at 98,587 (95% CI: 82,978 – 114,196), corresponding to 2.3% of the total adult female population aged 15 years and above in Rwanda. We estimated that in the City of Kigali, 5.3%, in the West Province, 2.2%, in the East and South province, 1.7% each, and in the North province 1.6% were FSW as % of adult female population 15 years of age and older who were living in Kigali and these Provinces.

We estimated that in the City of Kigali, 5.3%, in the West Province, 2.2%, in the East and South province, 1.7% each, and in the North province 1.6% were FSW as % of adult female population 15 years of age and older who were living in Kigali and these Provinces.

Conclusion: This was the first time that PNS was implemented as a PSE method in Rwanda, adding to the emerging tools that we have in the hard-to-reach PSE field. The PSE provides fundamental information to design, plan, and implement programs for FSW at the provincial level in Rwanda. Furthermore, these estimates will help to generate positive policy changes and to advocate for resources that will help in the effort to achieve a sustained HIV epidemic control in the country.

Key words: Female Sex Workers, Population Size Estimation, HIV, Privatize Network Sampling, Respondent Driven Sampling

Introduction

Globally, gay men and other men who have sex with men, sex workers, transgenders, people who inject drugs, and people in prisons and other closed settings are considered the five main key populations (KPs) that are particularly vulnerable to HIV and frequently lack adequate access to health services[1]. Some studies conducted on the HIV/AIDS epidemic have revealed the high burden of HIV infection among KPs.

The World Health Organization (WHO) highlights the need to focus on KP who are particularly vulnerable and disproportionately affected by HIV due to risk behaviors, marginalization, and structural factors such as stigma, discrimination, violence, human rights violations, and criminalization, which contribute to a lack of access to prevention and treatment services [2, 3] and hence become the key drivers of new HIV transmission in the general population. During 2022, in Eastern and Southern Africa, 54% of the total number of new HIV infections were reported among the general population, and the remainder were reported among KPs, with 13% of those reported among female sex workers (FSW) [4].

In Rwanda, FSWs are considered among key population groups for HIV prevention and treatment focus due to their high-risk behaviors for contracting and/or transmitting STIs/HIV, mainly because of multiple partners and a low rate of condom use. They are often stigmatized and marginalized and relatively disproportionately affected by HIV. Due to a continued observed high prevalence of HIV among FSW compared to the general population, FSWs are considered a persistent niche of HIV and are thus treated as the bridge of HIV infection to the general population [5-7]. The HIV prevalence among FSWs in Rwanda has exhibited a downward trend over the years[8]. In 2010, the HIV prevalence among FSW in Rwanda stood at 50.8%[5]. Subsequently, in 2015, it decreased to 45.8%[6], and the most recent survey conducted in 2019 indicates a further reduction to 35.5% [9].

Estimating the size of the population of FSW is important because it generates a denominator for program design, planning, and implementation. In addition, it informs interventions and public health initiatives and helps to determine the appropriate resource allocation, gaps and unmet needs to serve such community. It is sometimes difficult to survey them and obtain trustworthy estimates for population size, since being marginalized sometimes leads to their rare visibility in communities, thus becoming hard-to-reach groups.

Various methods have been used thus far to estimate the population sizes of hard-to-reach groups, each with its own set of strengths and drawbacks. Capture-recapture [10], multiplier [11], network scale-up [12], and successive sampling [13] approaches are all common. Unfortunately, there is no gold standard approach, and various methodologies frequently result in contradictory conclusions [14].

Respondent Driven Sampling (RDS)[15] has been proven to be able to penetrate the networks of hard-to-reach populations, assuming that members of the population are connected. Furthermore, capture-recapture (CRC) has been commonly used in epidemiology to estimate the size of hard-to-reach key populations targeted by health interventions for certain health conditions due to its mathematically grounded and defensible results[16-19]. To gain from the RDS and CRC strength features, a networked capture-recapture approach known as Privatized Network Sampling (PNS) was developed that is based on the same foundation but collects additional important information for the estimation of the size of the hard-to-reach population[20, 21].

Using the trust embedded in social ties has been shown to be a valuable technique for obtaining samples from hard-to-reach groups. Designs for tracking network links, such as respondent-driven sampling (RDS)[15, 22], have been widely used to study and draw inferences about hard-to-sample populations. One significant limitation of this method is that the degree time series contains only a small amount of information about population size. Privatized Network Sampling (PNS), population size estimation method was suggested for this study. This uses the network information collected within a bio-behavioral survey (BBS) that used RDS with further information collect on how individuals are networked to determine PSE.

In Rwanda, there have been three rounds of FSW PSE dated 2010[23], 2018[24] and 2022, all using Time Location Sampling (TLS). As the digital era emerges, with technologies reshaping and reorienting sex markets [25, 26], the use of venue-based sampling approaches might be missing a chunk of FSW who never congregate in the venues, including those practicing sex work at home (home-based) and those who get clients from internet platforms (internet-based).

Our aim was to estimate the size of FSW in the City of Kigali and in the four Provinces of Rwanda using Privatized Network Sampling (PNS) [27].

Materials and Methods

Study population

The PSE included self-identified FSW, any girl or woman aged 15 years and above whose main income was commercial sex work (i.e., exchanging money, goods, or services for sex). rephrase for clarity Furthermore scrutiny, the engagement in commercial sex work would have been in the last 12 months prior to the survey, providing informed consent to participate in the study and being able to communicate in any of the three official languages used in Rwanda (English, French, Kinyarwanda).

Study design and procedures

To inform the development of this estimation, a formative assessment (FA) was conducted. A group meeting that included implementing partners (IP), stakeholders, and FSWs was convened, and focus group discussions (FGD) were conducted. FSWs from 5 provinces in Rwanda came to Kigali for a one-day meeting on March 10th, 2023. The objectives of the FA included the identification of sociocultural factors limiting or facilitating access to FSW, assessing the feasibility of the planned method and procedures, and identifying barriers and strategies to overcome them.

This estimation utilized one single method of population size estimation, Privatized Network Sampling (PNS). This method utilized network data collected using the questions specifically developed for this purpose. Within a bio-behavioral survey (BBS) questionnaire that used RDS to sample FSW, more questions were added collecting information on the degrees at which FSW population are networked, hence used for size estimation purposes.

Data were collected countywide in 10 study sites, which included Gihundwe Health Center (HC), Kibuye HC, Gisenyi HC in the West; Gitarama HC and Rango HC in the South; Muhoza in the North; Mukarange HC and Nyagatare HC in the East; Remera HC and WE-ACT FOR HOPE CLINIC in the City of Kigali. Data collection was performed between May 8th, 2023, and June 24th, 2023.

The PNS sampling followed the Respondent Driven Sampling (RDS), which is a probability-based chain-referral sampling methodology used to sample FSWs for a biobehavioral survey (BBS). For the RDS process, initial survey participants ("seeds") were purposively recruited by the survey

team to start enrollment. Criteria to be a seed, one should have been engaged in commercial sex work at least 12 months prior to the estimation, well-connected within FSW social networks, well regarded by peers, able to communicate with data collectors, and supportive of estimation goals. Furthermore, three seeds by site were purposively recruited reflecting diversity in sociodemographic characteristics (e.g., age, sexual orientation and gender identity, education, area of residence, marital status, language, religion), HIV status, and affiliation with a KP organization or KP service provider.

Seeds and other subsequent recruiters were provided with a maximum of 3 coupons to distribute to their peers in their FSW social circle for recruitment. Instructions for peer recruitment using a recruitment process script were provided to seeds and participants by staff at the study site. When potential recruits came to the survey site, they were screened for eligibility and enrolled if they met the survey inclusion criteria and consented to participate; at this point, they were considered participants. After participating in the survey, these individuals were given their own recruitment coupons and asked to distribute them to their peers that they knew are FSW. This process continued until the target sample size and survey parameters were achieved.

However, RDS data contains limited information about participant's network. To collect major identifiable information about how individuals in the sample are related to one another, for each recruited respondent, using a cryptographic hash function, a hashed (anonymized) ID was created from the initials of the first and last name and the last 5 digits of the respondent's phone number using Tele funked coding [28].

Furthermore, each recruited respondent was asked how many of the total network size peers the recruit knows their name and phone numbers, and a hashed ID was also created for up to 5 peers in the respondent's personal network. If the respondent stated knowing 5 or fewer peers, a hashed ID was created for each of them. If the respondent knows more than 5 peers, then 5 peers were selected in a near-random fashion using an age-related selection process of peers with an age that was closest to the participant. The entered data (initials, last 5 digits of phone number) were not stored, and the hash ID was stored. The hashed ID could not be used to reconstruct the respondent's provided data. This information helped to evaluate the rate at which participants' networks contained other sampled participants.

Sample size and sampling

The sample size calculation was based on the FSW biobehavioral survey (BBS), which was sufficiently powered to estimate the provincial-level HIV. The sample size calculation was based on the province-specific prevalence of HIV among FSWs aged 15 years and above, estimated from the previous rounds of FSW BBS[9].

The design effect (Deff) for each province was estimated at 0.998 in East Province, 1.683 in West Province, 1.221 in North Province, 3.245 in South Province and 1.508 in Kigali City. The 95% Z score value was 1.96 with an alpha level of 0.05 (95% confidence), and the finite population correction was applied using the results of 2018 FSW population size estimation (PSE) [24]. As a result, the minimum sample size of 2,500 was estimated: East and South with 415, West with 623, North with 503 and City of Kigali with 544.

Data management

Data were collected electronically using an Open Data Kit (ODK)[29] installed on android tablets. All collected data were reviewed daily and checked for errors before submission. Daily, data from completed participant's questionnaires were electronically pushed to a password-protected database to ensure data safety.

Data quality checks were conducted regularly to ensure that high-quality data were generated. Mainly for PNS purposes, the RDS plot was run to view the recruitment graph and check if the tree matches what actually was happening in recruitment, checked for duplicated hashed IDs (duplicates are expected, but here, we checked that these are indeed unique individuals), checked for cases where a single subject reports the same hashed ID value for two of their contacts, and checked for subjects who report the exact same network contacts.

At the end of data collection, all study site-level data in a CSV format (Comma Delimited) were merged with coupon recruitment information from RDS to track for chain referral aspects.

Statistical analysis

A complete dataset was composed of key PNS variables, including subject ID (unique identifiers for individuals in the dataset), recruiter ID (unique identifier for the recruiter of the subject), subject hash ID (the privatized identifier for the subject), degree (the network size of the individual,

this excludes contacts for whom the individual does not know their identifiers), and contact hash IDs (privatized identifiers for each contact of the subject).

Each site-level dataset was converted into an RDS coupon data frame, which means that each recruiter is aligned alongside the corresponding direct recruits. With this data frame, each seed is regarded as the base of a tree that branches out as its recruits recruit more people, and each tree is its own sample.

Three estimators were considered, including *Cross-Sample Estimator,* which uses the overlap between each individual's friend group and the sampled individuals in other trees. The other estimator was *Cross-Alter Estimator,* which uses the overlap between each individual's friend group and friend groups from other sampled individuals who are in different trees. The third estimator was the *Cross-Network Estimator,* which combines the Cross-Sample and Cross-Alter Estimators. The intuition for PNS is "the rate at which subjects' networks contain other sampled subjects recruited by other seeds is related to population size."

All data analysis processes were performed using RStudio-2023.06.2-561 with the "RDS" and "*pnspop*" packages. Confidence intervals were calculated using the bootstrap process with the number of samples set to 10,000.

Site-level estimates were aggregated at the national level, then the pooled national estimates were distributed by province using proportions of FSW from existing program data. The analysis outputs include point estimates with corresponding 95% credible intervals. Finally, each estimate was adjusted for the proportion of FSW who did not have cell phones.

Ethical consideration

The survey was reviewed and approved by the Rwanda National Ethics Committee (RNEC). A written informed consent was obtained from the study participants to be part of the survey. Furthermore, a waiver of informed consent for participants aged 15 to 17 years was granted by RNEC. Data collection staff completed training on human subjects' research and signed a confidentiality agreement before the start of enrollment. All participants provided informed consent before enrollment in the survey. Participants were compensated with 5,000 Rwf (4.24 USD) for transportation costs and time during their first visit and 2,000 Rwf (1.70 USD) for the

second visit as compensation for time and 1,000 Rwf (0.85 USD) for each successful referral enrolled in the survey.

Prior to implementation, field staff received a one-week standardized training together in one site, followed by a half-day refresher training at their respective sites. These trainings focused on general knowledge of FSW, ethical issues in human subject research, and standard operating procedures for the RDS survey and PNS implementation.

Provide more details on the ethical considerations and procedures for obtaining informed consent from the study participants, especially those who are minors or vulnerable to exploitation and violence.

Results

In total, 30 FSWs were enrolled as seeds across 10 study sites countrywide to initiate referral chain recruitment. The maximum wavelength achieved during recruitment was 11 with a mode of 4. *Figure 1* below illustrates the RDS recruitment tree by province.

Figure 1: Recruitment tree by province, Rwanda FSW PNS 2023.

The highest proportion of FSW were estimated among those aged 30-39 years, with the smallest proportion among 15-19 age group (4.8%) (see *Table 1*)

Table 1: Social demographic characteristics of participants by province, Rwanda FSW PNS 2023.

		Province					
		North	East	West	City of Kigali	South	Overall
	N	Row % [95% CI]	Row % [95% CI]	Row % [95% CI]	Row % [95% CI]	Row % [95% CI]	Row % [95% CI]
**Age group**							
15 - 17	23	2.6 [1.4 - 4.8]	0.3 [0.1 - 1.5]	1.5 [0.5 - 4.1]	0.4 [0.1 - 1.7]	0.0 [0.0 - 0.2]	0.8 [0.5 - 1.3]
18 - 24	515	26.3 [22.1 - 30.9]	21.3 [16.5 - 27.1]	25.4 [20.8 - 30.7]	24.5 [18.3 - 31.9]	13.6 [9.6 - 19.1]	20.7 [17.9 - 23.9]
25 - 29	497	23.5 [19.4 - 28.0]	22.4 [17.3 - 28.5]	20.8 [16.8 - 25.5]	12.9 [9.1 - 18.0]	19 [14.3 - 24.7]	18.1 [15.7 - 20.8]
30 - 34	535	22.2 [18.1 - 26.9]	21.7 [16.6 - 27.9]	19.8 [15.9 - 24.3]	15.8 [12.3 - 20.2]	26.3 [20.2 - 33.5]	21.4 [18.6 - 24.6]
35 - 39	502	15.0 [11.9 - 18.9]	17.7 [13.4 - 22.9]	19.6 [15.6 - 24.3]	20.0 [15.6 - 25.1]	23.9 [18.3 - 30.6]	20.4 [17.7 - 23.4]
40+	439	10.5 [7.8 - 13.9]	16.6 [12.3 - 22.0]	12.9 [9.9 - 16.8]	26.4 [20.9 - 32.8]	17.1 [12.6 - 22.8]	18.6 [15.9 - 21.5]
**Current marital status**							
Single	1733	73.3 [68.5 - 77.5]	74.9 [69.0 - 80.0]	77.8 [73.0 - 81.9]	55.0 [48.3 - 61.5]	78.7 [72.4 - 83.9]	69.8 [66.5 - 72.9]
Married/Cohabitating	54	0.3 [0.0 - 2.4]	0.8 [0.3 - 2.5]	1.3 [0.3 - 5.0]	9.3 [6.6 - 13.0]	0.7 [0.2 - 2.6]	3.5 [2.5 - 4.8]
Divorced/Separated	633	24.5 [20.4 - 29.1]	22.0 [17.1 - 27.7]	16.4 [13.0 - 20.5]	27.8 [22.6 - 33.6]	16.8 [12.2 - 22.8]	22.0 [19.3 - 24.9]
Widow	88	1.9 [0.9 - 4.0]	2.3 [1.1 - 4.8]	4.2 [2.4 - 7.5]	7.9 [4.5 - 13.3]	3.5 [1.7 - 7.0]	4.6 [3.2 - 6.7]
Prefer not to answer	3	0	0	0.3 [0.1 - 1.4]	0	0.3 [0.0 - 2.1]	0.1 [0.0 - 0.7]
**Education level**							
None	490	16.7 [13.2 - 20.9]	25.9 [20.4 - 32.4]	22.9 [18.4 - 28.2]	16.5 [12.5 - 21.4]	18 [13.1 - 24.2]	18.0 [15.5 - 20.8]
Primary	1327	43.5 [38.5 - 48.5]	50.7 [44.1 - 57.2]	49.1 [43.7 - 54.5]	55.1 [48.4 - 61.7]	59.8 [52.6 - 66.6]	54.0 [50.5 - 57.6]
Secondary/vocational/higher education	662	39.8 [34.9 - 44.9]	23.4 [18.4 - 29.3]	24.5 [20.2 - 29.3]	28.4 [22.7 - 34.9]	20.5 [15.2 - 27.0]	27.1 [24.0 - 30.3]
Do not know/No answer	32	0	0	3.5 [1.9 - 6.4]	0	1.7 [0.6 - 4.8]	0.9 [0.4 - 1.9]
**Where do you usually meet or find clients?**							
Brothel/Guesthouse/Massage/Parlor	118	0.2 [0.0 - 1.1]	1.8 [0.6 - 5.1]	4.8 [3.2 - 7.0]	16.2 [12.2 - 21.3]	3.2 [1.5 - 6.5]	6.9 [5.4 - 8.9]
Hotel/Club/Bar/Restaurant	1110	47.8 [42.8 - 52.9]	54.6 [48.1 - 61.1]	58 [52.6 - 63.2]	36.1 [29.4 - 43.3]	75.5 [69.5 - 80.6]	55.3 [51.8 - 58.8]
Street/Park	720	22.2 [18.3 - 26.7]	7.5 [5.6 - 9.9]	21.2 [17.1 - 25.9]	34.5 [28.8 - 40.8]	8.3 [5.3 - 12.8]	20.3 [17.8 - 23.1]
Other public places	66	2.8 [1.3 - 6.2]	4.4 [2.1 - 9.0]	1.4 [0.6 - 3.0]	1.4 [0.6 - 3.2]	2.0 [1.0 - 4.0]	2.0 [1.4 - 3.0]
Internet, phone broker, escort agency	467	26 [21.7 - 30.8]	30.1 [24.4 - 36.6]	12.4 [9.3 - 16.3]	11.5 [8.0 - 16.3]	9.6 [6.6 - 13.7]	14.3 [12.3 - 16.6]
Other	29	1.0 [0.2 - 4.3]	1.6 [0.6 - 4.3]	2.2 [1.0 - 4.7]	0.2 [0.1 - 0.4]	1.5 [0.6 - 3.8]	1.0 [0.6 - 1.9]
Prefer not to answer	1	0	0	0.1 [0.0 - 0.9]	0	0	0.0 [0.0 - 0.1]
**Have a Sim card and cell phone**							
Yes	2002	75.2 [70.5 - 79.3]	95.9 [94.3 - 97.1]	76.1 [70.8 - 80.7]	86.6 [82.1 - 90.1]	74.0 [67.2 - 79.9]	79.4 [76.4 - 82.2]
No	509	24.8 [20.7 - 29.5]	4.1 [2.9 - 5.7]	23.9 [19.3 - 29.2]	13.4 [9.9 - 17.9]	26.0 [20.1 - 32.8]	20.6 [17.8 - 23.6]
**Has at least one peer's contact**							
Yes	1509	93.6 [88.3 - 96.6]	91.5 [89.2 - 93.4]	59.6 [53.5 - 65.5]	64.3 [56.6 - 71.4]	96.0 [94.5 - 97.1]	81.4 [78.0 - 84.4]
No	493	6.4 [3.4 - 11.7]	8.5 [6.6 - 10.8]	40.4 [34.5 - 46.5]	35.7 [28.6 - 43.4]	4.0 [2.9 - 5.5]	18.6 [15.6 - 22.0]
**Number of peers from whom contacts are available**							
One peer	188	3.7 [1.9 - 7.2]	2.4 [1.4 - 3.9]	15.7 [10.4 - 23.0]	37.3 [28.7 - 46.7]	1.8 [1.1 - 2.9]	12.9 [9.9 - 16.6]
Two peers	270	6.8 [4.5 - 10.1]	1.7 [0.9 - 3.3]	26.5 [20.9 - 33.0]	32.8 [25.6 - 41.0]	2.7 [1.8 - 4.1]	13.2 [10.9 - 16.0]
Three peers	548	36.6 [31.1 - 42.4]	95.6 [93.5 - 97.0]	37.4 [30.3 - 45.2]	16.6 [11.7 - 23.1]	4.1 [2.5 - 6.9]	21.3 [18.8 - 24.2]
Four Peers	128	16 [12.0 - 21.1]	0.2 [0.0 - 1.2]	9.9 [6.6 - 14.6]	7.1 [4.3 - 11.5]	5.8 [2.9 - 11.4]	8.1 [6.2 - 10.5]
Five peers	238	22.1 [17.6 - 27.4]	0.1 [0.0 - 0.3]	8.3 [5.3 - 12.9]	3.3 [1.6 - 6.6]	67.2 [59.3 - 74.3]	33.1 [28.7 - 37.8]
More than five peers	137	14.8 [11.3 - 19.1]	0.0 [0.0 - 0.1]	2.2 [1.0 - 4.6]	2.9 [1.3 - 6.3]	18.3 [12.7 - 25.7]	11.3 [8.7 - 14.6]

Regarding marital status, the majority, 69.8% (95% CI: 66.5-72.9), were single. An estimated 14.3% (95% CI: 12.3-16.6) of FSW meet clients using the internet, phone brokers, or escort agency (Table 1). Of all recruited FSW, 79.4% (95% CI: 76.4 -82.2) had a cell phone and a Sim card, and 81.4% (95% CI: 78.0 – 84.4) of them had at least nominated an FSW peer of whom she knows by name and has a telephone contact.

Table 2 presents both unadjusted and adjusted study site-level population size estimates by each estimator used.

		**Unadjusted Estimates**			**Adjusted Estimates**		
**Study site**	**Estimators**	PSE	Lower Bound	Upper Bound	PSE	Lower Bound	Upper Bound
GIHUNDWE HC	Cross-Sample	15,538	3,399	71,023	19,599	4,288	89,586
GISENYI HC	Cross-Sample	1,463	1,033	2,073	1,494	1,054	2,116
GITARAMA HC	Cross-Sample	6,256	2,884	13,571	7,839	3,613	17,005
KIBUYE HC	Cross-Sample	5,875	2,995	11,527	8,728	4,448	17,123
MUHOZA HC	Cross-Sample	104,226	41,554	261,421	140,369	55,964	352,075
MUKARANGE HC	Cross-Sample	1,021	773	1,349	1,371	1,038	1,811
NYAGATARE HC	Cross-Sample	3,905	1,945	7,842	3,905	1,945	7,842
RANGO HC	Cross-Sample	2,903	2,151	3,919	3,852	2,854	5,200
CITY OF KIGALI	Cross-Sample	11,314	8,029	15,942	14,414	10,229	20,310
GIHUNDWE HC	Cross-Alter	4,043	2,975	5,494	5,099	3,752	6,930
GISENYI HC	Cross-Alter	1,511	1,159	1,969	1,542	1,183	2,009
GITARAMA HC	Cross-Alter	6,006	3,830	9,418	7,526	4,799	11,801
KIBUYE HC	Cross-Alter	3,696	2,830	4,827	5,490	4,203	7,170
MUHOZA HC	Cross-Alter	27,472	21,272	35,479	36,999	28,649	47,783
MUKARANGE HC	Cross-Alter	354	226	556	476	303	746
NYAGATARE HC	Cross-Alter	10,166	5,196	19,892	10,166	5,196	19,892
RANGO HC	Cross-Alter	4,650	3,580	6,039	6,170	4,750	8,013
CITY OF KIGALI	Cross-Alter	18,457	13,978	24,373	23,514	17,807	31,051
GIHUNDWE HC	Cross-Network	5,479	3,996	7,512	6,911	5,040	9,476
GISENYI HC	Cross-Network	1,491	1,193	1,863	1,522	1,218	1,901
GITARAMA HC	Cross-Network	6,106	4,117	9,055	7,651	5,159	11,346
KIBUYE HC	Cross-Network	4,241	3,343	5,381	6,300	4,965	7,993
MUHOZA HC	Cross-Network	32,758	25,288	42,434	44,117	34,057	57,149
MUKARANGE HC	Cross-Network	597	454	785	801	610	1,053
NYAGATARE HC	Cross-Network	7,200	4,364	11,881	7,200	4,364	11,881
RANGO HC	Cross-Network	4,191	3,351	5,243	5,562	4,446	6,957
CITY OF KIGALI	Cross-Network	14,540	11,991	17,630	18,523	15,277	22,460

Site-level population size estimate variation was observed across estimators. The population size estimates produced by the cross-sample estimator were greater than those produced using the cross-Alter estimator at the Gihundwe HC, Kibuye HC, Muhoza HC, and Mukarange HC study sites. Population size estimates from Gisenyi HC and Gitarama HC remained almost the same across the two estimators. However, population size estimates produced by the cross-sample estimator were less than those produced using the cross-Alter estimator for the Nyagatare HC, City of Kigali, and Rango HC study sites. Site-level population size estimates produced using the cross-network estimator were observed to be between the estimates produced using cross-sample and cross-Alter estimators except for the Gisenyi HC study site. The same patterns were observed for both adjusted and unadjusted population size estimates; however, the differences are not statistically significant (see *Table 2)*.

Table 3 presents aggregated site-level population size estimates as a pooled estimate by the estimator used to produce the estimate.

Table 3: Overall Population Size Estimates by estimator, Rwanda FSW BBS 2023.

	**Unadjusted Estimates**			**Adjusted Estimates**		
**Estimators**	PSE	Lower Bound	Upper Bound	PSE	Lower Bound	Upper Bound
Cross-Sample	152,502	64,763	388,667	201,570	85,434	513,067
Cross-Alter	76,354	55,045	108,046	96,981	70,643	135,394
Cross-Network	76,603	58,097	101,782	98,587	82,978	114,196

Looking at the pooled population size estimate, the Cross-Sample estimator tends to produce larger estimates compared to the Cross-Alter and Cross-Network estimators considering both unadjusted and adjusted estimates. On the other hand, population size estimates produced using cross-Alter and cross-network estimators go hand in hand considering both adjusted and unadjusted estimates (see *Table 3)*.

Table 4 presents adjusted FSW population size estimates produced using the cross-network estimator, which combines both cross-sample and cross-Alter estimator features by administrative province.

Table 4: Cross-Network Estimator's Population Size Estimates by province, Rwanda FSW BBS 2023.		
**Province**	Consensus Population Size Estimate [95%CI]	*PSE as % of female aged 15+ years of the general population
North	11,317 [9,526 -13,109]	1.6% [1.3-1.9]
South	15,826 [13,320 -18,331]	1.7% [1,4-1.9
East	19,833 [16,693 -22,973]	1.7% [1.4-2.0]
West	20,593 [17,332 -23,853]	2.2% [1.8-2.5]
City of Kigali	31,018 [26,107- 35,929]	5.3% [4.5-6.1]
**Total**	**98,587 [82,978- 114,196]**	**2.3% [1.9-2.6]**

**Calculated based on the 5th Rwanda Housing and Population census, 2022[30].*

The national level FSW PSE was estimated at 98,587 (95% CI: 82,978 – 114,196), corresponding to 2.3% of the total adult female population aged 15 years and above in Rwanda. The highest FSW PSE was observed in the City of Kigali with 31,018 (95% CI: 26,107 – 35,929), followed by West province with 20,593 (95% CI: 17,332 – 23,853), the East province with 19,833 (95% CI: 16,693 – 22,973), and the South province with 15,826 (95% CI: 13,320 – 18,331). The lowest FSW population size was estimated in the North province with 11,317 (95% CI: 9,526 – 13,109).

Discussion

The national population size of female sex workers and sexually exploited minors aged 15 and above in Rwanda was estimated at 98,587 (95% CI: 82,978 – 114,196), corresponding to 2.3% of the total females aged 15 years and above in Rwanda based on the 5th Rwanda Housing and Population census, 2022[31]. The highest FSW concentration was found in the City of Kigali, with 5.3% [4.5 – 6.1] and the lowest in the North province with 1.6% [1.3 – 1.9] as % of female aged 15+ years of the general population.

Due to persistent stigma around sex work, FSWs tend to practice sex work in high secrecy and sometimes adopt emerging technologies for privacy. Rwanda has been experiencing a generalized HIV prevalence over the last decade, with a concentrated HIV epidemic among FSWs[9], where HIV prevalence is more than tenfold that of the general population[32], making them to be considered among key populations for HIV programs. Having an estimate of the population size of FSW would inform programs and interventions to better plan, monitor, and prevent disease spillovers into the general population.

To date, there have been four rounds of studies aimed at estimating the population size of FSW in Rwanda, with the commonality of all using Time Location Sampling (TLS) methodologies[23, 24, 33]. Capture-recapture, enumeration, and multiplier were listed among the methods used, with a commonly stated methodological limitation of inability to tackle within non-venue-based FSW and leading to potential underestimation of FSW. In contrast, in this survey we used PNS, a PSE that is capable of reaching different FSW subgroups, including non-venue-based subgroups [20], and produces financial resources effective and more credible inferences about population size, given that it is built into RDS. this was already described in the introduction.

The reported FSW population size estimate looks larger when compared with previously estimated size of FSW population in Rwanda. The variances between the current FSW PSE and the previous ones can be more potentially attributed to the methodological capability to reach non-venue based FSWs around the country. The National HIV annual report 2022-23 reported a total number of 60,460 FSW identified during the reporting year period in the HIV program[34]. Believing that the program reported number is not exhaustive and recognizing the limitations associated with program data, including the possible inability to deidentify individual-level data, give more confidence in the PSE resulted from the current study.

Include more details on the potential sources of bias and error in the population size estimation, such as the assumptions of the privatized network sampling method, the quality and completeness of the network data, and the representativeness and diversity of the seeds and recruits.

One important limitation associated to the use of PNS to estimate the population size in our context, is related to the coverage of SIM cards and cell phones among FSW within the survey sample, which was used to produce hashed ids to uniquely identify overlaps (alters) in the tracing network sampling. Access to mobile technology varies among individuals, and not all FSWs may possess a SIM card or a functioning cell phone. Furthermore, the estimates from this study might have been affected by those FSW who decide not to provide FSW phone numbers, the data collection tool used that did enable to delimit participants only for the province where the survey was being implemented, and the social desirability bias of the BBS.

To minimize the effects of the listed limitations on the estimates, the study has adjusted the estimates to account for the proportion of those FSW who did not have SIM card and cell phones. In addition, to have provincial-level estimates, we assumed that the provincial-level distribution of FSW population reported by HIV program in its' 2022-2023 HIV annual report remains the same for the current FSW population size estimation, therefore the pooled estimate was proportionally distributed across provinces using the percent proportion distribution from the annual HIV report 2022-2023.

In conclusion, the population size estimate derived from this study serves to inform targeted interventions, resource allocation, advocacy efforts, resources mobilization, and policy development aimed at improving access to preventive, care and treatment services for FSW in Rwanda. Additionally, this is the first time that PNS is implemented for the estimation of the FSW population size estimate in Rwanda, adding to the emerging tools that we have in the hard-to-reach PSE field.

Emphasize the key messages or takeaways of the study and provide some directions or priorities for future research, such as the areas that need further investigation, the methods or data that need refinement or innovation, or the stakeholders or partners that need engagement or support

Declaration of competing interest

The authors have no conflicts of interest to disclose.

Acknowledgments

We express our gratitude to all the study participants and the team of co-investigators who helped make this work possible. Additionally, we would like to convey our sincere gratitude to the CDC team for their crucial technical support during the entire study's implementation.

References

1. Organization, W.H., *Focus on key populations in national HIV strategic plans in the WHO African Region*. 2018, World Health Organization. Regional Office for Africa.
2. UNAIDS, *UNAIDS Global AIDS Update*. 2023.
3. WHO, *FOCUS ON KEY POPULATIONS IN NATIONAL HIV STRATEGIC PLANS IN THE AFRICAN REGION*. 2018.
4. UNAIDS, *UNAIDS Global AIDS Update* 2022.
5. Mutagoma, M., et al., *High HIV prevalence and associated risk factors among female sex workers in Rwanda.* International journal of STD & AIDS, 2017. **28**(11): p. 1082-1089.
6. Mutagoma, M., et al., *Syphilis and HIV prevalence and associated factors to their co-infection, hepatitis B and hepatitis C viruses prevalence among female sex workers in Rwanda.* BMC infectious diseases, 2017. **17**(1): p. 1-9.
7. Nsanzimana, S., et al., *Prevalence and incidence of HIV among female sex workers and their clients: modelling the potential effects of intervention in Rwanda.* BMJ global health, 2020. **5**(8): p. e002300.
8. Mutagoma, M., et al., *Sexual risk behaviors and practices of female sex workers in Rwanda in over a decade, 2006–2015.* International journal of STD & AIDS, 2018. **29**(13): p. 1316-1323.
9. Rwanda Biomedical Center/Institute of HIV/AIDS, D.P., *INTEGRATED BEHAVIORAL AND BIOLOGICAL SURVEILLANCE SURVEY AMONG FEMALE SEX WORKERS IN RWANDA, 2019*. 2019.
10. Chao, A., et al., *The applications of capture-recapture models to epidemiological data.* Statistics in medicine, 2001. **20**(20): p. 3123-3157.
11. Johnston, L.G., et al., *Incorporating the service multiplier method in respondent-driven sampling surveys to estimate the size of hidden and hard-to-reach populations.* Sexually transmitted diseases, 2013. **40**(4): p. 304-310.
12. Maltiel, R., et al., *Estimating population size using the network scale up method.* The annals of applied statistics, 2015. **9**(3): p. 1247.
13. Kim, B.J. and M.S. Handcock, *Population size estimation using multiple respondent-driven sampling surveys.* Journal of Survey Statistics and Methodology, 2021. **9**(1): p. 94-120.
14. Vadivoo, S., et al., *Appropriateness and execution challenges of three formal size estimation methods for high-risk populations in India.* Aids, 2008. **22**: p. S137-S148.
15. Heckathorn, D.D., *Respondent-driven sampling: a new approach to the study of hidden populations.* Social problems, 1997. **44**(2): p. 174-199.
16. Van Hest, N., *Capture-recapture methods in surveillance of tuberculosis and other infectious diseases*. 2007.
17. Des Jarlais, D., et al., *Using dual capture/recapture studies to estimate the population size of persons who inject drugs (PWID) in the city of Hai Phong, Vietnam.* Drug and Alcohol Dependence, 2018. **185**: p. 106-111.
18. Vuylsteke, B., et al., *Estimating the number of female sex workers in Côte d'Ivoire: results and lessons learned.* Tropical Medicine & International Health, 2017. **22**(9): p. 1112-1118.
19. Hook, E.B. and R.R. Regal, *Capture-recapture methods in epidemiology: methods and limitations.* Epidemiologic reviews, 1995. **17**(2): p. 243-264.
20. Fellows, I.E., *Estimating Population Size from a Privatized Network Sample.* Journal of Survey Statistics and Methodology, 2022. **10**(5): p. 1346-1369.
21. Handcock, M.S., *Discussion of "Adaptive and Network Sampling for Inference and Interventions in Changing Populations" by Steven K. Thompson.* Journal of Survey Statistics and Methodology, 2017. **5**(1): p. 29-33.

22. Mwaniki, S.W., P.M. Mugo, and T. Palanee-Phillips, *Project BESPOKE (Integrated Bio-Behavioral Assessment of HIV and STI Among Young Tertiary Student Men Who Have Sex With Men in Nairobi, Kenya): A Respondent-Driven Sampling Survey Protocol.* Frontiers in Public Health, 2021: p. 1452.
23. Mutagoma, M., et al., *Estimation of the size of the female sex worker population in Rwanda using three different methods.* International Journal of STD & AIDS, 2015. **26**(11): p. 810-814.
24. Musengimana, G., et al., *Female sex workers population size estimation in Rwanda using a three-source capture− recapture method.* Epidemiology & Infection, 2021. **149**.
25. Sanders, T., et al., *Internet sex work: Beyond the gaze*. 2018: Springer.
26. Jones, A., *Sex work in a digital era.* Sociology Compass, 2015. **9**(7): p. 558-570.
27. Khan, B., et al., *One-step estimation of networked population size: Respondent-driven capture-recapture with anonymity.* Plos one, 2018. **13**(4): p. e0195959.
28. Dombrowski, K., et al., *Estimating the size of the methamphetamine-using population in New York City using network sampling techniques.* Advances in applied sociology, 2012. **2**(4): p. 245.
29. Hartung, C., et al. *Open data kit: tools to build information services for developing regions*. in *Proceedings of the 4th ACM/IEEE international conference on information and communication technologies and development*. 2010.
30. (NISR), N.I.o.S.o.R., *5th Rwanda Population and Housing Census (PHC)*. 2022.
31. Rwanda, N.I.o.S.o., *Fifth Population and Housing Census - 2022*. 2022: https://www.statistics.gov.rw/datasource/fifth-population-and-housing-census-2022.
32. Nsanzimana, S., et al., *HIV incidence and prevalence among adults aged 15-64 years in Rwanda: Results from the Rwanda Population-based HIV Impact Assessment (RPHIA) and District-level Modeling, 2019.* International Journal of Infectious Diseases, 2022. **116**: p. 245-254.
33. Rwanda Biomedical Center/Institute of HIV/AIDS, D.P., et al., *Estimating the size of populations through a household survey*. 2012, RBC/IHDPC, SPF, UNAIDS, and ICF International Calverton, Maryland, USA.
34. (RBC), R.B.C., *HIV Annual Report, Rwanda*. 2022-2023, Rwanda Biomedical Center (RBC).

Click here to access/download;Figure;RDS recruitment trees by province.pdf

RDS recruitment trees by province

Eastern Province recruitment tree, Rwanda FSW BBS 2023.

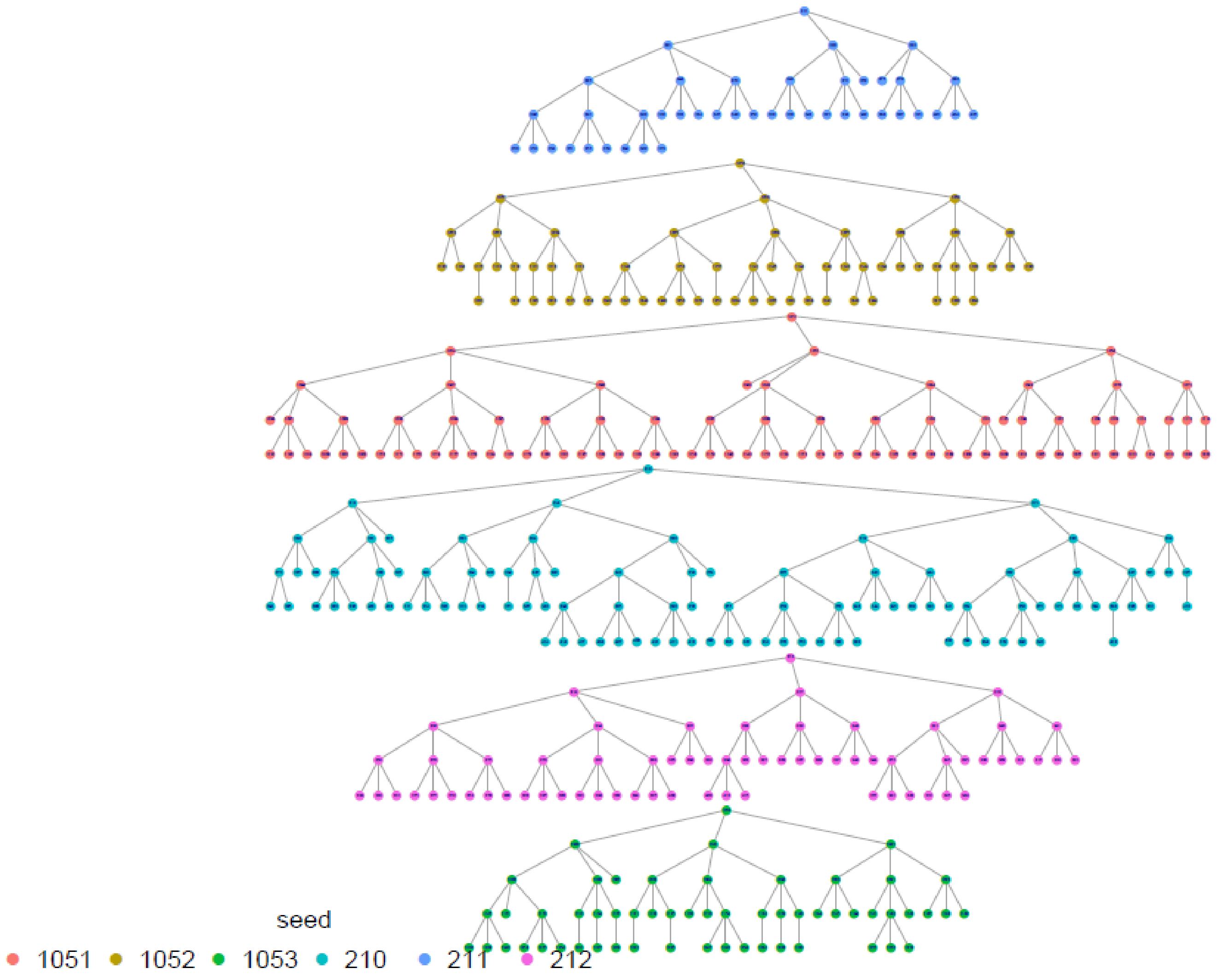

City of Kigali recruitment tree, Rwanda FSW BBS 2023.

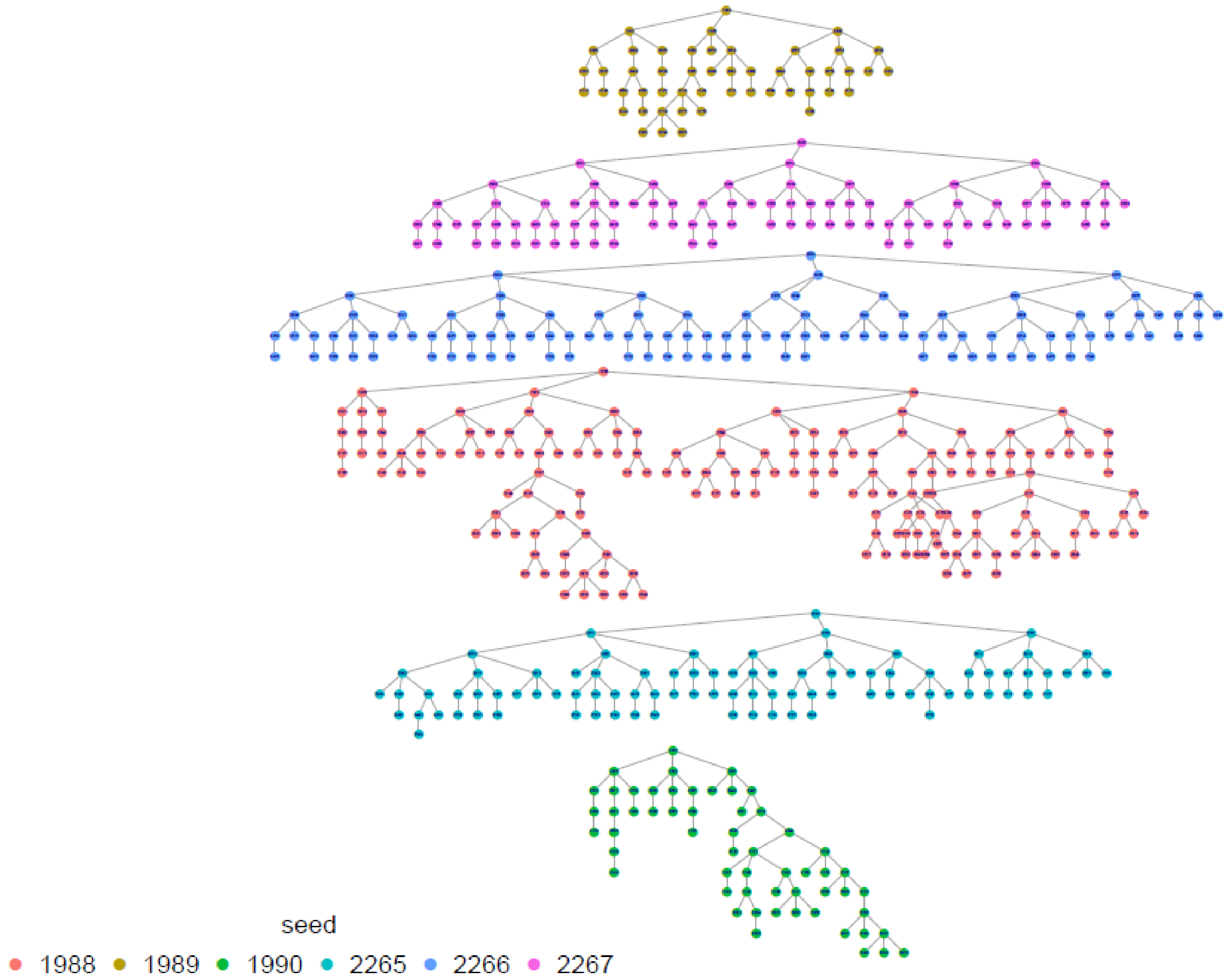

Northern province recruitment tree, Rwanda FSW BBS 2023.

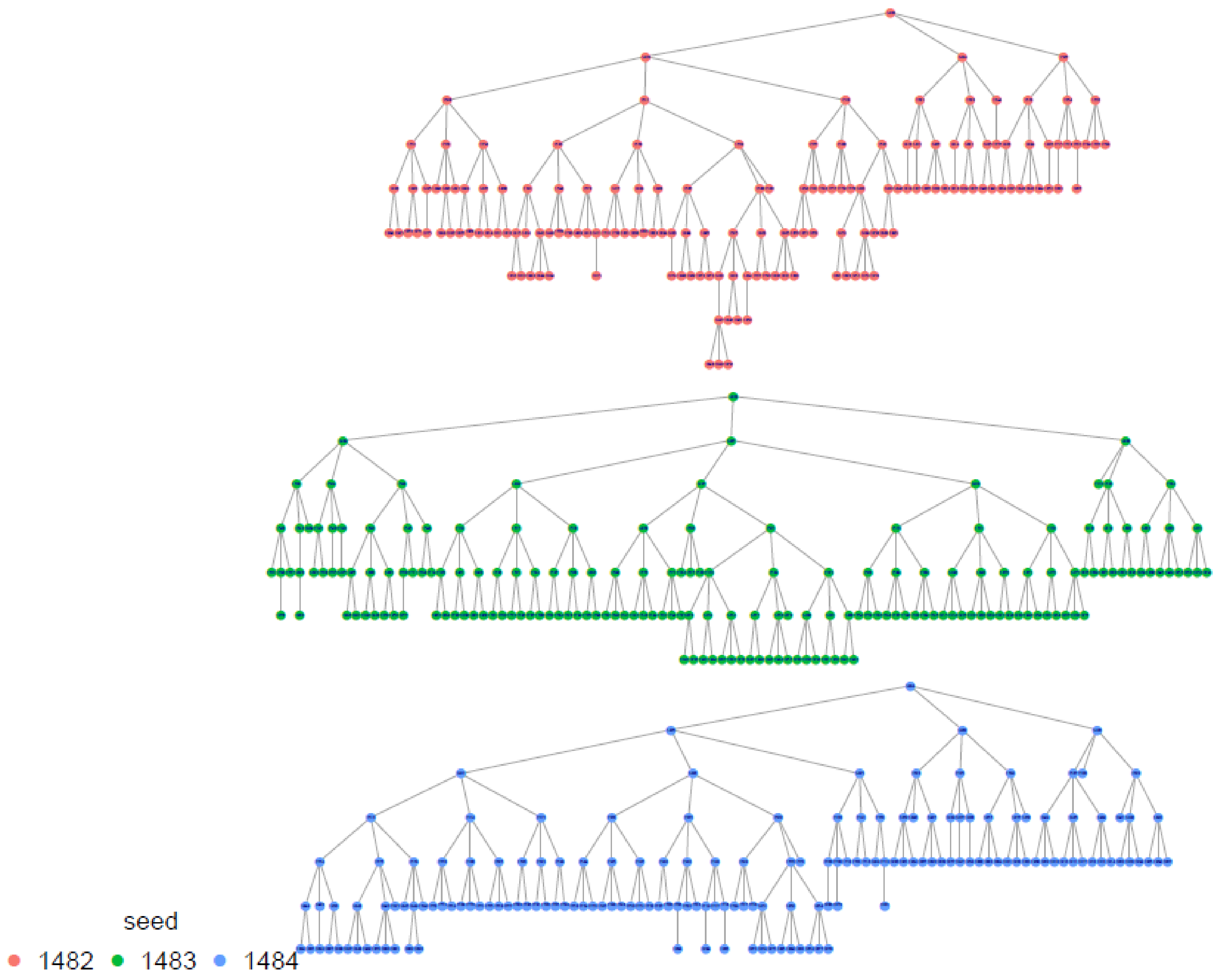

Southern province recruitment tree, Rwanda FSW BBS 2023.

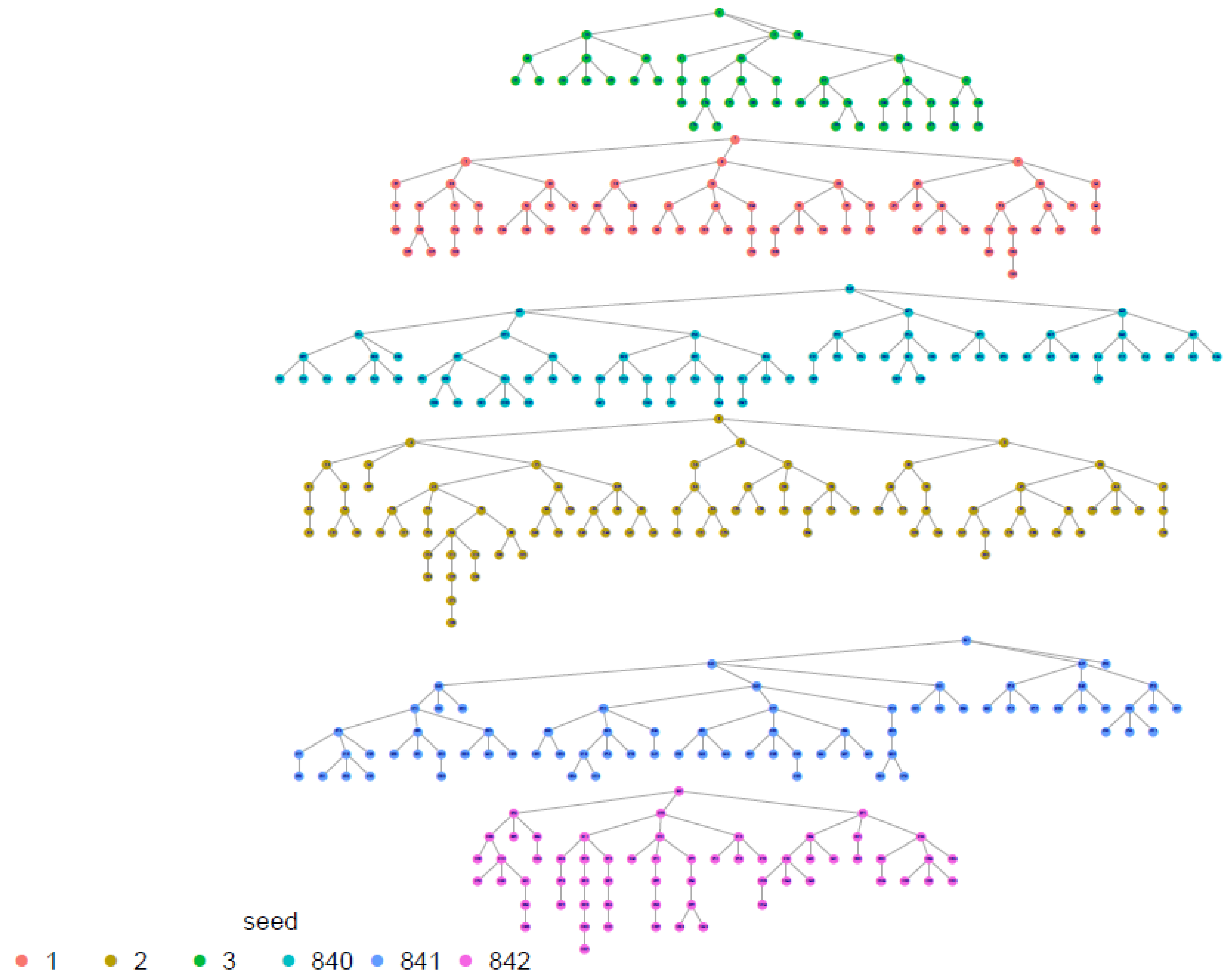

Western province recruitment tree, Rwanda FSW BBS 2023.

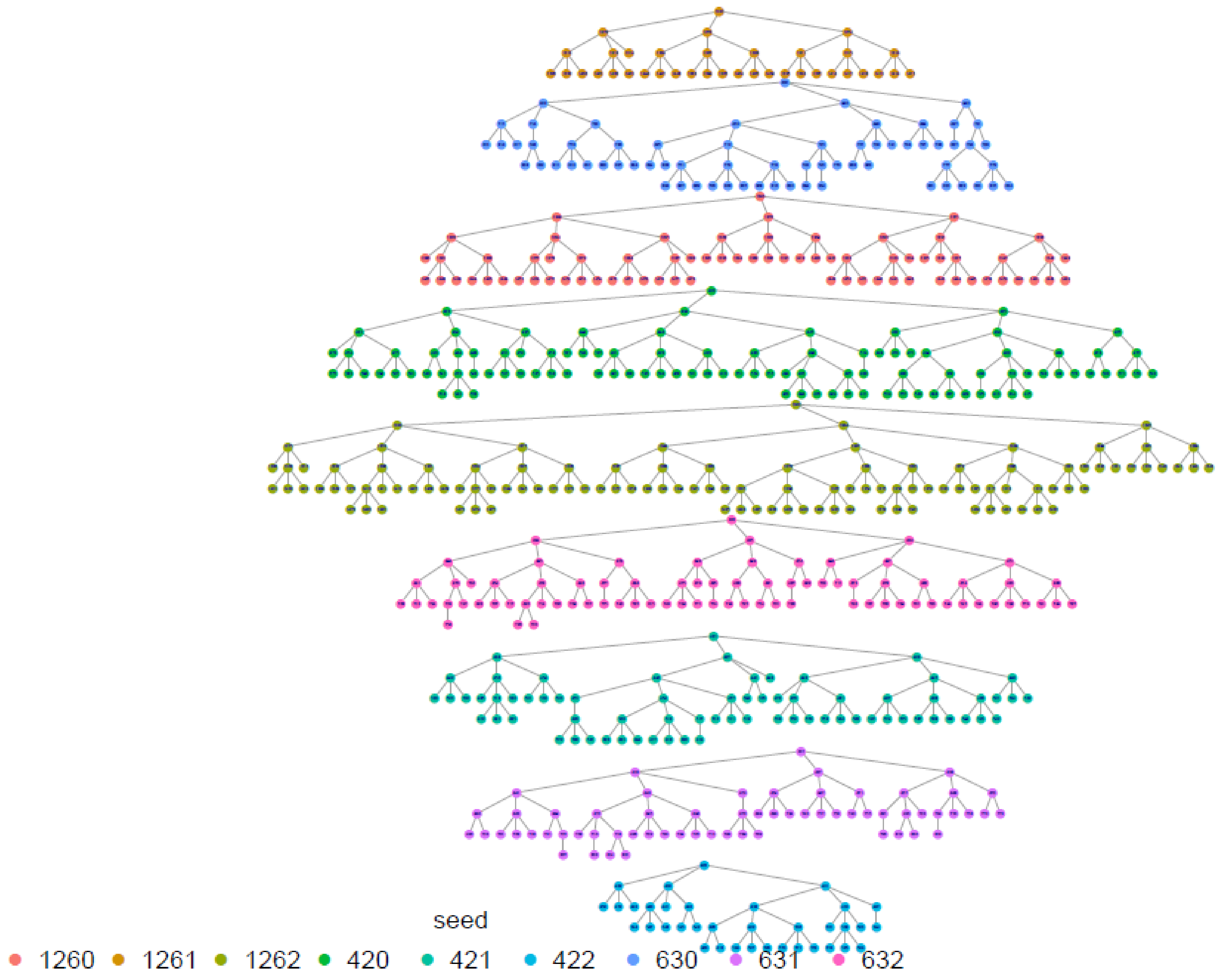

Supplement: Attachment — Submitted filename: Research article with comments.pdf [file pone.0329772.s001.pdf]
