# Supplementary material for: Estimating the size of hard to sample populations: A comprehensive study on female sex workers and sexually exploited minors in Rwanda using privatized network sampling in 2023
Source: PLoS One. 2025 Aug 21;20(8):e0329772. doi: 10.1371/journal.pone.0329772 (PMC12370040; doi:10.1371/journal.pone.0329772)
Supplement: Attachment — Submitted filename: PONE-D-23-42748R2_Reviewer Feedback .pdf [file pone.0329772.s005.pdf]

| Estimating the Size of Hard to Sample Populations: A Comprehensive Study on Female Sex Workers and Sexually Exploited Minors in Rwanda using Private Network Sampling in 2023. | PONE-D-23-42748R2 |
|---|---|

## Abstract

General comments:

Overall aim, design and key findings well-articulated

Detailed comments:

## Introduction

General comments:

The introduction is well-structured and provides a clear context for the study; however, there are a few places where it might be strengthened to increase its depth, clarity, and connection with the goals of the research.

Detailed comments:

**Limited coverage of sexually exploited minors**

- The inclusion of sexually exploited minors seems secondary and underexplored, it is only stated at the very end, with no explanation or context provided earlier. Their inclusion should be made clearer and related to the study objectives. Consider introducing sexually exploited minors as a secondary but equally critical focus earlier in the text.
- More background on their unique vulnerabilities, significance in HIV prevention efforts, and challenges in PSE would provide balance.

**Limited discussion of existing sampling methods' drawbacks:**

- Even though the introduction notes the shortcomings of the current approaches such as RDS, CRC, and TLS, it should give concrete instances in which these approaches have fallen short or produced inconsistent results, especially when it comes to FSWs. This would justify why PNS is necessary.
- Consider describing the challenges with the current approaches by giving specific examples or studies demonstrating the shortcomings of TLS, RDS, or other methods in accurately estimating FSW or KP population sizes.

**Insufficient background on PNS**

- PNS is introduced as an approach for this study but its fundamental workings, advantages over other methods are not thoroughly explained. Providing more context about why PNS is particularly suitable for this population would strengthen its justification. Highlighting its specific advantages, novelty, and applicability to the digital and home-based FSW subgroups can justify its use in this study.

**Weak/fragmented justification for researching digital and home-based FSWs**

- The introduction discusses the growth of digital and home-based sex work in passing but doesn't go into detail on how big of a change this is in Rwanda or around the world.
- More evidence or examples of how the digital era has changed sex work would put in context the study's focus on previously unrepresented subgroups.

**Inadequate explanation of the study's importance**

- Although program design, planning, and resource allocation are mentioned in the introduction as reasons for population size estimation (PSE), there is no clear link made between these and the broader implications for HIV prevention, policy-making, or service provision.
- Think about relating the study's findings to broader implications for public health, policy, and resource allocation, especially in terms of HIV prevention and treatment strategies.

**Stronger research gap statement is required.**

- The study's significance and novelty are not explicitly stated. For instance, why is this study so necessary at this time, and how does it overcome the shortcomings of previous FSW PSE in Rwanda?

Clearly state how this study closes a knowledge gap or overcomes the shortcomings of earlier PSE research conducted in Rwanda and around the world.

## Materials and methods

Detailed comments:

**Study population**

**Definition and inclusion criteria ambiguity**

- The term *"self-identified FSW"* is subjective and could lead to underrepresentation or overrepresentation. There is no mention of strategies to validate self-identification. Clearly define *"self-identified FSW"* and describe validation measures to avoid misclassification.
- Even though non-binary or transgender people are involved in sex work and more vulnerable, they are not included in the population. Consider including provisions for non-binary or transgender individuals and discuss their representation in the study.

**Formative Assessment**

**Representativeness concerns**

- The FA included only 10 FSWs, a small and potentially unrepresentative sample. While diversity in characteristics was attempted, it may not capture the full spectrum of FSW experiences across provinces.
- The selection process for these participants is unclear, raising concerns about potential bias.

**Limited documentation of sociocultural insights**

- One of the FA objectives was to identify socio-cultural factors that influence FSW. Nothing is stated on how these factors were considered in study design and logistics. Provide specific examples of socio-cultural insights and explicitly describe how these informed study design and logistics.

**Privatized Network Sampling (PNS)**

**Limited justification for PNS approach**

- Although PNS is mentioned as an improvement over RDS, its specific advantages in the context of this population are not clearly highlighted. Consider providing a thorough explanation of PNS, emphasizing why it is suitable for the Rwandan FSW context.
- Potential drawbacks of PNS, like biases brought forth the network connectivity or participant errors in reporting peers, are not discussed. Discuss any possible prejudices or restrictions with the PNS approach and how they were mitigated.

**Confidentiality and anonymization concerns**

The description of hashing lacks sufficient detail to assess its robustness and alignment with best practices for data anonymization.

**Sampling and recruitment process**

**Potential recruitment bias**

- Seeds were purposively selected, which might introduce selection bias. While diversity was considered, there is no mention of how representative the seeds were of the broader FSW population.
- Recruitment relied on social networks, potentially excluding isolated or marginalized FSWs, such as those not connected to larger networks.
- There is need to explain extra steps used to guarantee the representativeness of isolated or hidden FSWs and justify the selection of seeds

**Sample framework**

**Justification for sample size**

- The methodology provides detailed calculations but does not explain doesn't describe why a design effect (Deff) was selected or how provincial differences influenced study design. Describe the selection of design effect values and the implications of provincial variations.
- The finite population correction is mentioned but its effects on accuracy and variability are not discussed Explain how the finite population correction is applied and its relevance to the study objectives.

**Data collection challenges**

**Limited discussion of logistical challenges**

- Practical challenges, such as participant mobility or regional variations in stigma, are not addressed. These could impact data collection and generalizability. Discuss logistical challenges (e.g. stigma, mobility, literacy) and strategies used to overcome them.

**Unclear approach for non-participants in personal networks**

- The study assumes participants know and can report on their peers. Those who are isolated or in hidden subgroups may be underrepresented. Explain how isolated or less-connected FSWs were accounted for in the recruitment and sampling process.

**Data management**

**Incompleteness of recruitment data validation**

- While recruitment graphs were checked, a thorough explanation of how abnormalities or discrepancies (such as duplicate hashed IDs or identical network contacts) were fixed is not given.
- The process of merging site-level data with RDS coupon information could benefit from more transparency, particularly in how recruitment chain inconsistencies were addressed. Improve documentation of data validation processes, especially when it comes to fixing duplicates and network anomalies.

**Statistical analysis**

**Estimator selection and bias mitigation**

- While the Cross-Network Estimator is reported to have reduced volatility, the justification for its preference over others is not clearly articulated. An exploration of estimator-specific biases and contextual applicability would strengthen the methodology.
- There is limited explanation of how differences between site-level and national estimates were accounted for beyond proportional adjustments using existing program data.
- Give a clear explanation of the selection criteria for the Cross-Network Estimator as the main approach and discuss how it applies to FSWs in Rwanda

**Exclusion of FSW without cell phones**

- Although adjustments for FSWs without cell phones are stated, but the methodology for determining the proportion of such individuals is unclear. This exclusion could lead to underrepresentation of vulnerable subgroups. Targeted sampling or additional data could improve adjustments for subpopulations without mobile phones or those not included in network sample.

**Provincial-level variations**

- While data are aggregated nationally, variations in recruitment success, network sizes, and subpopulation characteristics across provinces are not adequately explored. Analysis at the provincial level may provide insightful information and enhance local program design. Include analysis at the site and province levels to identify regional variations and inequalities in the population

## Results

### General comments:

The findings are clearly outlined.

## Discussion

### Detailed comments:

**Acknowledgment of limitations**

- While limitations (e.g., SIM card access, social desirability bias, and methodological assumptions) are noted, their potential quantitative impact on the estimates is not deeply analysed. For example, how might the lack of SIM cards influence the credibility intervals?
- Quantify the potential biases introduced by limitations like SIM card access and proportional distribution

**PNS Assumptions and justification**

- PNS assumptions, such as randomness of connections and sufficient recruitment waves, are briefly acknowledged but not critically evaluated against observed recruitment dynamics. Were there any biases or recruitment bottlenecks?
- The methodological advantages of PNS are highlighted, but the discussion lacks a nuanced exploration of where it might still fall short compared to other methodologies, such as network-based approaches not relying on technology.
- Critically evaluate the PNS fundamental presumptions and how well they apply to the context in Rwanda.

**Insights on hard-to-reach subgroups**

- While the study highlights its ability to capture non-venue-based FSW, the discussion does not look into specific findings about these subgroups or how their inclusion changes programmatic priorities.
- The challenges faced in reaching sexually exploited minors, as a subgroup distinct from adult FSWs, are largely unaddressed.
- Highlight specific insights gained about non-venue-based FSWs and sexually exploited minors and their implications for interventions.

**Implications and future directions**

- The implications for program design and policy are general and repetitive. There is limited specific guidance on how these findings should shape targeted HIV interventions or broader health programs for FSWs.
- Future research recommendations focus on smaller-area estimations but fail to suggest methodological innovations or longitudinal follow-ups to track population dynamics over time.
- Recommend broader methodological innovations and longitudinal studies for future research.
