# Supplementary material for: Estimating the size of hard to sample populations: A comprehensive study on female sex workers and sexually exploited minors in Rwanda using privatized network sampling in 2023
Source: PLoS One. 2025 Aug 21;20(8):e0329772. doi: 10.1371/journal.pone.0329772 (PMC12370040; doi:10.1371/journal.pone.0329772)
Supplement: Attachment — Submitted filename: PONE-D-23-42748_R3.pdf [file pone.0329772.s006.pdf]

Estimating the Size of Hard to Sample Populations: A Comprehensive Study on Female Sex Workers and Sexually Exploited Minors in Rwanda using Private Network Sampling in 2023.

Thank you for the opportunity to review this manuscript. I am the 3rd reviewer of this article and I will be assessing how well the authors have incorporated feedback from the comments and edits suggested by Reviewers 1 and 2.

The article discusses a study aimed at estimating the population size of female sex workers (FSW) in Rwanda, a group at high risk for HIV and other sexually transmitted infections (STIs). The study used Privatized Network Sampling (PNS), a method incorporating network data from a bio-behavioral survey, to estimate the number of FSWs and sexually exploited minors. The findings suggest that the national FSW population is approximately 98,587, which represents 2.3% of the adult female population aged 15 and older in Rwanda. The proportion of FSWs varied across regions, with the highest estimate in the City of Kigali (5.3%) and lower estimates in other provinces.

The study highlights the first use of PNS as a population size estimation method in Rwanda, offering valuable data for HIV/STI prevention program planning. These estimates are intended to guide program design, support policy changes, and advocate for resources to achieve sustained HIV epidemic control in the country.

<u>Overall article feedback and response to reviewers and journal editors comments:</u>

1) *Reference list*

I can see that the following changes have been made in the Reference List. Please try to specifically list all changes in response to reviewer's comments for ease of review.

In the most up-to-date version of the manuscript I see that the following reference:

2. UNAIDS, Global AIDS Update. 2023.
3. UNAIDS, Global AIDS Update 2022.

Replace these earlier references:

2. HIV/AIDS, J.U.N.P.o., The path that ends AIDS: 2023 UNAIDS global AIDS update. 2023
3. Payagala, S. and A. Pozniak, The global burden of HIV. Clinics in Dermatology, 2024. 42(2): p. 119- 127

2) *The survey was reviewed and approved by the Rwanda National Ethics Committee (RNEC).' Thus, protocol ID is highly needed.*

To which the authors replied: Action taken: Thanks for the comment, the protocol ID number of the protocol covering the survey is added under “Ethical consideration” section (page #9) as it appears on the approval letter secured from Rwanda National Ethics Committee (ID: IRB 00001497 of IORG0001100.

I can see that the protocol ID is indicated.

* typeset	
**Ethics Statement**<br><br>Enter an ethics statement for this submission. This statement is required if the study involved:<br><br>• Human participants<br>• Human specimens or tissue<br>• Vertebrate animals or cephalopods<br>• Vertebrate embryos or tissues<br>• Field research	The survey was reviewed and approved by the Rwanda National Ethics Committee. Data collection staff completed training on human subjects' research and signed a confidentiality agreement before the start of enrollment. All participants provided informed consent before enrollment in the survey. Protocol ID number: IRB 00001497 of IORG0001100.

Powered by Editorial Manager® and ProduXion Manager® from Aries Systems Corporation

3) *Naming requirements*

This review agrees with the renamed title. The article follows PLOS One style guidelines as indicated at https://journals.plos.org/plosone/s/submission-guidelines

4) *Data availability*

Data availability statement is made

**Describe where the data may be found in full sentences. If you are copying our sample text, replace any instances of XXX with the appropriate details.**	The data underlying the results presented in the study are available for researchers who meet set criteria upon request and approval by RBC (data access requests are sent at: 'info@rbc.gov.rw').

5) *Reference list*

Best practice is to include the DOI when one is available. A few entries to update:

Reference #7:

Mutagoma, M., et al., Sexual risk behaviors and practices of female sex workers in Rwanda in over a decade, 2006–2015. International journal of STD & AIDS, 2018. 29(13): p. 1316-1323 https://doi.org/10.1177/0956462418785297

Reference #28:

Hartung, C., et al. Open data kit: tools to build information services for developing regions. in Proceedings of the 4th ACM/IEEE international conference on information and communication technologies and development. 2010.
https://doi.org/10.1145/2369220.236923

6) *Greater clarity in manuscript titleI*

Authors have used the reviewer's suggested title.

7) *Reviewer suggested that providing some context and motivation for why estimating the size of FSW population is important for HIV prevention and control in Rwanda.*

Authors responded and included an updated sentence: "Having population size estimates of the HIV-mostly affected population, FSW in this case provides the basis for determining the denominators to assess HIV program performance towards national and global targets of controlling the HIV epidemic among the FSW population."

This reviewer would suggest that the authors move up the following paragraph to the section after the paragraph that starts, "Various have been used thus…" as it more clearly positions why the methods used by the study team are needed:
"In Rwanda, there have been three rounds of FSW PSE dated 2010[22], 2018 [23]and 2022, all using Time Location Sampling (TLS). As the digital era emerges, with technologies reshaping and reorienting sex markets[24, 25], the use of venue-based sampling approaches might be missing a chunk of FSW who never congregate in the venues, including those practicing sex work at home (home-based) and those who get clients from internet platforms (internet-based)."

8) *Reviewer suggested explicitly stating aims of the study*

Authors responded and have done so.

9) *Reviewer suggested editing for repetition*

Authors have addressed this concern.

10) *Reviewer suggested providing a clear statement of the research question or objective at the end of the introduction, and how it relates to the existing knowledge gap or research need.*

Authors have addressed this concern.

11) *Reviewer suggested grounding work in hypothesis*

Authors have addressed this concern.

12) *Reviewer suggested greater description of the ethical considerations when working with a highly marginalized population*

Authors have included a robust section on ethical concerns.

13) *Reviewer suggested more details were needed for the formative assessment*

Authors have addressed this concern.

14) *Reviewer suggested greater detail on statistical analysis*

Authors have addressed this concern.

15) *Reviewer suggested greater attention to result interpretation*

Authors have addressed this concern and this section has greater contextual and interpreted details.

16) Reviewer suggested more attention to implications, future work, and limitations
Authors have substantially revised this section

<u>Additional items for consideration</u>

1) In the opening sentence, please revise the term “transgenders” to “transgender people” or “transgender individuals.” Transgenders is an adjective, and when used as a noun it needs to apply to a person.
2) Please ensure that statements are supported by references. In the second line the authors state, “ Some studies conducted on the HIV/AIDS epidemic have revealed the high burden of HIV infection among KPs” but do not provide any evidence or reference to studies.
    a) Another example is “They are often stigmatized and marginalized and relatively disproportionately affected by HIV” again without references.
    b) Another example is “Respondent Driven Sampling (RDS)[15] has been proven to be able to penetrate the networks of hard-to-reach populations, assuming that members of the population are connected.” References are needed to support the use of this method for sex workers or justification on why this method should be applied to sex workers.
3) Some sentences could be rewritten for greater clarity
    a) As an example, “"Surveying this group and obtaining reliable population estimates can be challenging, as their marginalization often results in limited visibility within communities, making them hard-to-reach populations" is more concise and direct than “It is sometimes difficult to survey them and obtain trustworthy estimates for population size, since being marginalized sometimes leads to their rare visibility in communities, thus becoming hard-to-reach groups.”
4) Please provide examples and directly link them to the statement in text “Using the trust embedded in social ties has been shown to be a valuable technique for obtaining samples from hard-to-reach groups.”
5) Throughout the article the terms “female” and “woman” are used interchangeably. Best practice is to consider sex and gender as distinct variables and be explicit about these choices. You should distinguish between **sex** and **gender** when biological and social factors have different implications for the study or issue at hand. In contexts where both biological and societal factors are important, it’s necessary to carefully consider how both interact and how each contributes to the outcomes being measured. For HIV-related research arguments can be made to consider these as separate variables if the topic of investigation is related to some biological-specific detail. In HIV prevention research, sex is an essential variable to examine because of its role in biological susceptibility, transmission dynamics, and prevention outcomes. Recognizing and accounting for sex differences ensures that HIV prevention strategies are scientifically

sound, personalized, and effective across different populations, particularly those with varying biological, social, and behavioral risks.

6) Please revise this sentence for readability: “The PSE included self-identified FSW, any girl or woman aged 15 years and above whose main income was commercial sex work (i.e., exchanging money, goods, or services for sex). Furthermore scrutiny, the engagement in commercial sex work would have been in the last 12 months prior to the survey, providing informed consent to participate in the study and being able to communicate in any of the three official languages used in Rwanda (English, French, Kinyarwanda).”
    a) Suggestion: "The PSE included self-identified female sex workers (FSW), defined as girls or women aged 15 years and older whose primary source of income was commercial sex work (i.e., exchanging sex for money, goods, or services). Additionally, participants were required to have engaged in commercial sex work at least once in the 12 months prior to the survey, provided informed consent to participate, and be able to communicate in one of Rwanda's three official languages (English, French, or Kinyarwanda)."
7) Formatting: please do a once-over; there are a few sections where the font style seems to change and the spacing after the period changes. Some sentences start with a lower-case letter … some words are not complete “e.g. cros instead of cross”
8) Please clarify what it meant to “get confident” in this paragraph “For the study design and procedure’s theme, we got confident in the proposed method (PNS), decided to use name initials and the last 5 digits of one’s phone number combination to form the unique identification number of participants, informed study site preparation and setting, informed coupon (invitation) design, learned that there is a need for the engagement of local government, and received insights on the content development of training materials for data collectors.”
    a) This reviewer suggests breaking this into two sentences for greater clarity
9) There is reference to activities (HIV testing?) being done but this is not included in the study procedures, “ Children <18 years of age identified as being engaged in sex work, trafficked, or victim of violence, received a special post HIV test counseling and were referred for appropriate services to ensure their protection and well-being.”
10) Suggest renaming Table 2 for greater clarity. Tables should be able to stand-alone from the manuscript and impart information on what is presented.
11) Suggest finding a different way to express “vibrating areas” such as “busy economic and social locations”
12) This needs explanation: “sometimes adopt emerging technologies for privacy.”
13) I would suggest that the authors consider another limitation to the study which may underestimate the size of the population. Sex workers were recruited whose main income was “commercial sex work.” Many sex workers globally are involved in sex work, sometimes in a “part-time” or “as needed” fashion. It would be important to consider the exclusion of these people in the estimates.
