# Supplementary material for: Estimating the size of hard to sample populations: A comprehensive study on female sex workers and sexually exploited minors in Rwanda using privatized network sampling in 2023
Source: PLoS One. 2025 Aug 21;20(8):e0329772. doi: 10.1371/journal.pone.0329772 (PMC12370040; doi:10.1371/journal.pone.0329772)
Supplement: Attachment — Submitted filename: Response to Reviewers_r3.docx [file pone.0329772.s008.docx]

**Responses to ACADEMIC EDITOR’s comments (in Yellow)**

**Reviewer #2:**

1. In the opening sentence, please revise the term “transgenders” to “transgender people”

or “transgender individuals.” Transgenders is an adjective, and when used as a noun it

needs to apply to a person.

**Action taken**: Thanks for the correction, “transgenders” was replaced by “transgender people” under introduction section, first sentence.

1. Please ensure that statements are supported by references. In the second line the

authors state, “ Some studies conducted on the HIV/AIDS epidemic have revealed the

high burden of HIV infection among KPs” but do not provide any evidence or reference

to studies.

**Action taken**: Thanks for spotting this, a reference is added ( ref [2]) to support the statement.

a) Another example is “They are often stigmatized and marginalized and relatively

disproportionately affected by HIV” again without references.

**Action taken**: Thanks for the comment, the highlighted area seems to be a long sentence, but at the end of the sentence, we provided to references to support the statements (ref [1,2]).

b) Another example is “Respondent Driven Sampling (RDS)[15] has been proven to

be able to penetrate the networks of hard-to-reach populations, assuming that

members of the population are connected.” References are needed to support

the use of this method for sex workers or justification on why this method should

be applied to sex workers.

Thank you for this important comment. We agree that further clarification and support are needed for the use of Respondent Driven Sampling (RDS) in the context of sex workers. In response, we have revised the manuscript to include references that document the successful application of RDS among sex worker populations. For example, RDS has been widely used in studies involving sex workers due to their often hidden and stigmatized status, which limits the effectiveness of traditional probability sampling methods (Johnston et al., 2013; Heckathorn, 1997; Baral et al., 2012). These studies demonstrate that RDS can effectively reach diverse segments within this population through peer recruitment mechanisms. We have added these citations to the revised manuscript and clarified the rationale for selecting RDS in this context

3. Some sentences could be rewritten for greater clarity

a) As an example, “"Surveying this group and obtaining reliable population

estimates can be challenging, as their marginalization often results in limited

visibility within communities, making them hard-to-reach populations" is more

concise and direct than “It is sometimes difficult to survey them and obtain

trustworthy estimates for population size, since being marginalized sometimes

leads to their rare visibility in communities, thus becoming hard-to-reach groups.”

Thanks for the comment and suggestion, the sentence is revised for clarity and reads as: "Surveying this group and obtaining reliable population estimates is challenging, as their marginalization often results in limited visibility within communities, making them a hard-to-reach population."

2. Please provide examples and directly link them to the statement in text “Using the trust

embedded in social ties has been shown to be a valuable technique for obtaining

samples from hard-to-reach groups.”

Thank you for the helpful suggestion. We have revised the sentence to include concrete examples of hard-to-reach populations where leveraging trust within social networks has enabled successful sampling, and we have added appropriate citations to support this statement (Heckathorn, 1997; Johnston & Sabin, 2010; Baral et al., 2012).

3. Throughout the article the terms “female” and “woman” are used interchangeably. Best

practice is to consider sex and gender as distinct variables and be explicit about these

choices. You should distinguish between **sex** and **gender** when biological and social

factors have different implications for the study or issue at hand. In contexts where both

biological and societal factors are important, it’s necessary to carefully consider how

both interact and how each contributes to the outcomes being measured. For

HIV-related research arguments can be made to consider these as separate variables if

the topic of investigation is related to some biological-specific detail. In HIV prevention

research, sex is an essential variable to examine because of its role in biological

susceptibility, transmission dynamics, and prevention outcomes. Recognizing and

accounting for sex differences ensures that HIV prevention strategies are scientifically sound, personalized, and effective across different populations, particularly those with

varying biological, social, and behavioral risks.

Thank you for highlighting the importance of distinguishing between sex and gender. We have revised the manuscript to use these terms appropriately, referring to biological factors as “sex” and social factors as “gender,” and clarified their relevance where both are applicable. Changes made are reflected as track changes in the revised manuscript.

4. Please revise this sentence for readability: “The PSE included self-identified FSW, any

girl or woman aged 15 years and above whose main income was commercial sex work

(i.e., exchanging money, goods, or services for sex). Furthermore scrutiny, the

engagement in commercial sex work would have been in the last 12 months prior to the

survey, providing informed consent to participate in the study and being able to

communicate in any of the three official languages used in Rwanda (English, French,

Kinyarwanda).”

a) Suggestion: "The PSE included self-identified female sex workers (FSW), defined

as girls or women aged 15 years and older whose primary source of income was

commercial sex work (i.e., exchanging sex for money, goods, or services).

Additionally, participants were required to have engaged in commercial sex work

at least once in the 12 months prior to the survey, provided informed consent to

participate, and be able to communicate in one of Rwanda's three official

languages (English, French, or Kinyarwanda)."

Thank you for the suggestion. We have revised the sentence for clarity and readability as follows:

“The PSE included self-identified female sex workers (FSWs), defined as girls or women aged 15 years and above whose primary source of income was commercial sex work (i.e., exchanging sex for money, goods, or services). Eligibility also required engagement in commercial sex work within the past 12 months, ability to communicate in one of Rwanda’s official languages (English, French, or Kinyarwanda), and provision of informed consent.”

5. Formatting: please do a once-over; there are a few sections where the font style seems

to change and the spacing after the period changes. Some sentences start with a

lower-case letter … some words are not complete “e.g. cros instead of cross”

Thank you for the helpful feedback. We have conducted a thorough review of the manuscript to ensure consistent formatting, correct font style, proper spacing, and accurate spelling and capitalization throughout.

6. Please clarify what it meant to “get confident” in this paragraph “For the study design and

procedure’s theme, we got confident in the proposed method (PNS), decided to use

name initials and the last 5 digits of one’s phone number combination to form the unique

identification number of participants, informed study site preparation and setting,

informed coupon (invitation) design, learned that there is a need for the engagement of

local government, and received insights on the content development of training materials

for data collectors.” a) This reviewer suggests breaking this into two sentences for greater clarity

Thank you for the suggestion. We have revised the sentence for clarity and readability. Specifically, we replaced "got confident" with a clearer phrase and divided the content into two sentences to better convey the sequence and nature of insights gained during the study design phase.

7. There is reference to activities (HIV testing?) being done but this is not included in the

study procedures, “ Children <18 years of age identified as being engaged in sex work,

trafficked, or victim of violence, received a special post HIV test counseling and were

referred for appropriate services to ensure their protection and well-being.”

We appreciate the reviewer’s observation. The statement regarding HIV testing was part of the broader serosurveillance activities conducted alongside the population size estimation (PSE). However, since this manuscript focuses solely on the PSE component, we have removed this reference to avoid confusion and ensure clarity and relevance to the study objectives.

8. Suggest renaming Table 2 for greater clarity. Tables should be able to stand-alone from

the manuscript and impart information on what is presented.

Thank you for the suggestion. We have revised the title of Table 2 to improve clarity and ensure it can stand alone. The corrected title now reads: “Unadjusted and Adjusted Study Site-Level Population Size Estimates of Female Sex Workers by Estimation Method.”

9. Suggest finding a different way to express “vibrating areas” such as “busy economic and

social locations”

Thank you for pointing this out. We agree that “vibrating areas” might be unclear and have revised the sentence for better clarity.

10. This needs explanation: “sometimes adopt emerging technologies for privacy.”

Thank you for your comment. We agree that the phrase needed clarification. We have revised the sentence to provide a clearer explanation of what is meant by "emerging technologies for privacy."

13. I would suggest that the authors consider another limitation to the study which may

underestimate the size of the population. Sex workers were recruited whose main

income was “commercial sex work.” Many sex workers globally are involved in sex work,

sometimes in a “part-time” or “as needed” fashion. It would be important to consider the

exclusion of these people in the estimates.

Thank you for the thoughtful suggestion. This sentence is added: Acknowledging this limitation, we recognize that our estimates may underrepresent the full population of sex workers by excluding individuals who engage in sex work part-time or intermittently and do not consider it their main source of income.

**Reviewer #3:**

**Introduction**

General comments:

The introduction is well-structured and provides a clear context for the study; however, there are a few places where it might be strengthened to increase its depth, clarity, and connection with the goals of the research

Detailed comments:

**Limited coverage of sexually exploited minors**

- The inclusion of sexually exploited minors seems secondary and underexplored, it is only stated at the very end, with no explanation or context provided earlier. Their inclusion should be made clearer and related to the study objectives. Consider introducing sexually exploited minors as a secondary but equally critical focus earlier in the text.
- More background on their unique vulnerabilities, significance in HIV prevention efforts, and challenges in PSE would provide balance.

Thank you for this important observation. We have revised the manuscript to introduce sexually exploited minors under introduction section as well as under ethical considerations. To address this concern raised, the following paragraph was added:

“*Sexually exploited minors represent a particularly vulnerable subgroup within the broader population of individuals engaged in sex work, yet they are often underrepresented in research due to ethical, legal, and methodological challenges [**10**,* *11**]. Their involvement in commercial sex is typically driven by coercion, poverty, or exploitation, placing them at heightened risk of HIV infection and other adverse health outcomes [**12**]. Recognizing this, our study included sexually exploited minors, this refers to females aged 15 to 17 years engaged in sex work”*

**Limited discussion of existing sampling methods' drawbacks:**

- Even though the introduction notes the shortcomings of the current approaches such as RDS, CRC, and TLS, it should give concrete instances in which these approaches have fallen short or produced inconsistent results, especially when it comes to FSWs. This would justify why PNS is necessary.
- Consider describing the challenges with the current approaches by giving specific examples or studies demonstrating the shortcomings of TLS, RDS, or other methods in accurately estimating FSW or KP population sizes.

We appreciate the reviewer’s insightful comment regarding the limited discussion of existing sampling methods and the need to justify the use of Privatized Network Sampling (PNS). In response, we have revised the Introduction section to incorporate a more detailed discussion of the limitations associated with methods such as Time-Location Sampling (TLS), Respondent-Driven Sampling (RDS), Capture-Recapture (CRC), the multiplier method, and the Network Scale-Up Method (NSUM). Specifically, we now include examples of how these approaches have produced inconsistent or incomplete estimates among female sex workers (FSWs), often due to barriers related to stigma, limited-service contact, or assumptions about social network structures. To address these challenges, we justify the use of PNS, which has been demonstrated to enhance privacy and confidentiality in network reporting, making it well-suited for hard-to-reach and highly stigmatized populations. These revisions clarify the rationale for our methodological choice and align with the reviewer’s recommendations.

**Insufficient background on PNS**

- PNS is introduced as an approach for this study but its fundamental workings, advantages over other methods are not thoroughly explained. Providing more context about why PNS is particularly suitable for this population would strengthen its justification. Highlighting its specific advantages, novelty, and applicability to the digital and home-based FSW subgroups can justify its use in this study

Thank you for your comment. We agree that providing sufficient background on Privatized Network Sampling (PNS) is important to justify its use. As noted, the previously addressed comment regarding the limitations of existing methods and the rationale for selecting PNS also encompasses this concern. In response, we have expanded the Introduction section to explain the core principles of PNS, its comparative advantages—such as enhanced privacy protection and improved reach to stigmatized, digitally active, or home-based subgroups of female sex workers (FSWs)—and its suitability for this context. We have also included relevant citations to guide readers seeking more detailed information on the PNS methodology.

**Weak/fragmented justification for researching digital and home-based FSWs**

- The introduction discusses the growth of digital and home-based sex work in passing but doesn't go into detail on how big of a change this is in Rwanda or around the world.
- More evidence or examples of how the digital era has changed sex work would put in context the study's focus on previously unrepresented subgroups.

Thank you for your valuable feedback. In response, we have strengthened the Introduction section by elaborating on the significance of digital and home-based sex work, both globally and in the Rwandan context. We now discuss how the proliferation of mobile technology and internet access has transformed the sex work landscape, allowing many FSWs to operate independently, outside of traditional venues, and often in more discreet or home-based settings. This shift poses unique challenges for conventional sampling and outreach methods, making it critical to develop and apply approaches—like Privatized Network Sampling (PNS)—that can effectively reach these underrepresented subgroups. We have also included supporting evidence and citations to underscore the global trend and contextual relevance.

**Inadequate explanation of the study's importance**

- Although program design, planning, and resource allocation are mentioned in the introduction as reasons for population size estimation (PSE), there is no clear link made between these and the broader implications for HIV prevention, policy-making, or service provision.
- Think about relating the study's findings to broader implications for public health, policy, and resource allocation, especially in terms of HIV prevention and treatment strategies

We appreciate the reviewer’s observation regarding the need to clarify the broader importance of population size estimation. In response, we have revised the introduction to explicitly link PSE to its implications for HIV prevention, public health planning, policy-making, and service provision. Relevant citations have also been added to support this justification.

**Stronger research gap statement is required.**

- The study's significance and novelty are not explicitly stated. For instance, why is this study so necessary at this time, and how does it overcome the shortcomings of previous FSW PSE in Rwanda? Clearly state how this study closes a knowledge gap or overcomes the shortcomings of earlier PSE research conducted in Rwanda and around the world.

We appreciate the reviewer’s suggestion and have revised the introduction to clearly articulate the significance and novelty of the study. Specifically, we now emphasize that this study addresses known limitations of prior PSE exercises in Rwanda, including underestimation observed when comparing earlier PSE results with program data. Additionally, we highlight the importance of conducting repeated PSEs over time to reflect dynamic shifts in the FSW population and context—such as mobility, changes in operational typologies, and digital migration. By applying a novel approach and targeting underrepresented subgroups like home-based and internet-based sex workers.

**Materials and methods**

Detailed comments:

**Study population**

**Definition and inclusion criteria ambiguity**

- The term *“self-identified FSW”* is subjective and could lead to underrepresentation or overrepresentation. There is no mention of strategies to validate self-identification. Clearly define *“self-identified FSW”* and describe validation measures to avoid misclassification.
- Even though non-binary or transgender people are involved in sex work and more vulnerable, they are not included in the population. Consider including provisions for non-binary or transgender individuals and discuss their representation in the study.

We appreciate the reviewer’s thoughtful feedback regarding the definition and inclusion criteria. To reduce ambiguity and potential misclassification, we revised the terminology from “self-identified FSW” to “peer-identified FSW,” clarifying that participants were identified through peer networks, but enrollment required the individual's voluntary disclosure of engagement in sex work. This reflects ethical research principles, acknowledging that participation must be based on informed consent and self-disclosure, regardless of external perceptions. Additionally, we clarified that participants were those assigned female at birth to eliminate confusion regarding the inclusion of transgender or non-binary individuals. While we recognize the heightened vulnerability of transgender and non-binary sex workers, their exclusion in this study was due to the specific scope and design.

**Formative Assessment**

**Representativeness concerns**

- The FA included only 10 FSWs, a small and potentially unrepresentative sample. While diversity in characteristics was attempted, it may not capture the full spectrum of FSW experiences across provinces.
- The selection process for these participants is unclear, raising concerns about potential bias.

Thank you for the valuable feedback. In response, we have clarified that the 10 FSWs who participated in the Formative Assessment (FA) were purposively selected with the support of key stakeholders who work closely with FSW communities. At this stage, the goal was not to achieve representativeness but rather to obtain diverse perspectives to inform the study design and implementation. As the FA aimed to gather foundational qualitative insights, prioritizing diversity in age, location, and working modality was more appropriate than statistical representativeness. We have revised the manuscript to reflect this rationale more clearly.

**Limited documentation of sociocultural insights**

- One of the FA objectives was to identify socio-cultural factors that influence FSW. Nothing is stated on how these factors were considered in study design and logistics. Provide specific examples of socio-cultural insights and explicitly describe how these informed study design and logistics.

Thank you for the valuable feedback. In response, we have clarified that the 10 FSWs who participated in the Formative Assessment (FA) were purposively selected with the support of key stakeholders who work closely with FSW communities. At this stage, the goal was not to achieve representativeness but rather to obtain diverse perspectives to inform the study design and implementation. As the FA aimed to gather foundational qualitative insights, prioritizing diversity in age, location, and working modality was more appropriate than statistical representativeness. We have revised the manuscript to reflect this rationale more clearly.

**Privatized Network Sampling (PNS)**

**Limited justification for PNS approach**

- Although PNS is mentioned as an improvement over RDS, its specific advantages in the context of this population are not clearly highlighted. Consider providing a thorough explanation of PNS, emphasizing why it is suitable for the Rwandan FSW context.
- Potential drawbacks of PNS, like biases brought forth the network connectivity or participant errors in reporting peers, are not discussed. Discuss any possible prejudices or restrictions with the PNS approach and how they were mitigated.

We appreciate the reviewer’s insightful comments. The revised introduction now provides a more detailed justification for the use of Privatized Network Sampling (PNS), highlighting its advantages in reaching diverse and often hidden subgroups of FSWs, particularly in the Rwandan context. We also incorporated a discussion on the limitations of existing methods (e.g., RDS, TLS, CRC) with specific examples, and clarified how PNS overcomes some of their challenges. Furthermore, potential limitations of PNS are discussed in the discussion section.

**Confidentiality and anonymization concerns**

The description of hashing lacks sufficient detail to assess its robustness and alignment with best practices for data anonymization.

Thank you for highlighting this important aspect. We have revised the manuscript to include a clearer and more detailed description of the hashing procedure used for anonymizing participant identifiers. Specifically, we have clarified how the method aligns with established practices for data privacy and confidentiality, as supported by recent literature. The added paragraph ensures transparency in our approach and demonstrates the robustness of our anonymization method.

**Sampling and recruitment process**

**Potential recruitment bias**

- Seeds were purposively selected, which might introduce selection bias. While diversity was considered, there is no mention of how representative the seeds were of the broader FSW population.
- Recruitment relied on social networks, potentially excluding isolated or marginalized FSWs, such as those not connected to larger networks.
- There is need to explain extra steps used to guarantee the representativeness of isolated or hidden FSWs and justify the selection of seeds

To address concerns about recruitment bias in our respondent-driven sampling (RDS) approach, we added a paragraph in the manuscript that clarifies that seeds were purposively selected based on FA findings in a way that reflects diversity across key characteristics such as age, geographic location, and sex work modalities. This approach aligns with best practices in respondent-driven sampling (RDS), where purposive seed selection is standard to initiate recruitment chains that can reach various subgroups within hidden populations. We also implemented steps to enhance representativeness, including monitoring recruitment chains, introducing new seeds as needed, and engaging community organizations to reach isolated or marginalized FSWs. These efforts aimed to minimize bias and ensure broad inclusion, reflecting lessons learned from prior RDS studies, corresponding citations are included.

**Sample framework**

**Justification for sample size**

- The methodology provides detailed calculations but does not explain doesn't describe why a design effect (Deff) was selected or how provincial differences influenced study design. Describe the selection of design effect values and the implications of provincial variations.
- The finite population correction is mentioned but its effects on accuracy and variability are not discussed Explain how the finite population correction is applied and its relevance to the study objectives.

Thank you for the comment. We have clarified the rationale behind the selected design effect values, which reflect provincial variations in network dynamics, and explained the application and relevance of the finite population correction in improving sample accuracy—these updates have been incorporated into sample size and sampling section.

**Data collection challenges**

**Limited discussion of logistical challenges**

- Practical challenges, such as participant mobility or regional variations in stigma, are not addressed. These could impact data collection and generalizability. Discuss logistical challenges (e.g. stigma, mobility, literacy) and strategies used to overcome them.

Thank you for your insightful comment. We have added a paragraph at the end of study design section that discusses key logistical challenges encountered during data collection, including issues related to participants' access to phones and the difficulties in obtaining accurate peer identifiers due to lack of official names for hash id construction. We also detailed the practical strategies we used to overcome these barriers and ensure data consistency.

**Unclear approach for non-participants in personal networks**

- The study assumes participants know and can report on their peers. Those who are isolated or in hidden subgroups may be underrepresented. Explain how isolated or less-connected FSWs were accounted for in the recruitment and sampling process.

Thank you for this important observation. This concern has been addressed in our response to the earlier comment on "potential recruitment bias" under the study design and procedures section. In the context of RDS, we recognize the potential for underrepresentation of isolated or less-connected individuals. To mitigate this, we strategically selected diverse seeds across geographic locations and subgroups, including web-based and home-based FSWs, with input from key stakeholders who are familiar with harder-to-reach members of the FSW population during FA. This approach increased the likelihood that subsequent waves of recruitment would extend into more hidden sub-networks. Furthermore, we continued recruitment chains until equilibrium was reached and monitored network sizes to assess representativeness and saturation across FSW subgroups. These measures helped improve inclusion of isolated FSWs within the RDS framework.

**Data management**

**Incompleteness of recruitment data validation**

- While recruitment graphs were checked, a thorough explanation of how abnormalities or discrepancies (such as duplicate hashed IDs or identical network contacts) were fixed is not given.
- The process of merging site-level data with RDS coupon information could benefit from more transparency, particularly in how recruitment chain inconsistencies were addressed. Improve documentation of data validation processes, especially when it comes to fixing duplicates and network anomalies.

Thank you for your valuable comment. We have now clarified the data management and validation procedures in the revised manuscript. Specifically, we expanded on how daily data monitoring and review of recruitment chains (trees) guided real-time feedback and corrective actions in the field. We also described the use of cross-validation documents, and the systematic processes used to resolve inconsistencies such as duplicate hashed IDs or discrepancies in network ties.

**Statistical analysis**

**Estimator selection and bias mitigation**

- While the Cross-Network Estimator is reported to have reduced volatility, the justification for its preference over others is not clearly articulated. An exploration of estimator-specific biases and contextual applicability would strengthen the methodology.
- There is limited explanation of how differences between site-level and national estimates were accounted for beyond proportional adjustments using existing program data.
- Give a clear explanation of the selection criteria for the Cross-Network Estimator as the main approach and discuss how it applies to FSWs in Rwanda

Thank you for your helpful suggestions. We have revised the manuscript to better articulate the rationale for selecting the Cross-Network Estimator. Specifically, we added a summary of recent evidence showing that the Cross-Network Estimator consistently outperforms other estimators by minimizing both bias and variance. We also clarified that the Cross-Network Estimator’s use of information from both sampled and nominated peers makes it especially suitable for hidden populations like FSWs in Rwanda. Additionally, the national estimate was derived by proportionally aggregating site-level estimates using existing program data to reflect the distribution of FSWs across provinces.

**Exclusion of FSW without cell phones**

- Although adjustments for FSWs without cell phones are stated, but the methodology for determining the proportion of such individuals is unclear. This exclusion could lead to underrepresentation of vulnerable subgroups. Targeted sampling or additional data could improve adjustments for subpopulations without mobile phones or those not included in network sample.

Thank you for this important observation. We have addressed this concern in the revised manuscript by clarifying the methodology used to account for female sex workers (FSWs) without personal cell phones. Specifically, the proportion of FSWs without phones was calculated based on direct field reports from survey teams and incorporated into the final population size estimation. As discussed in the earlier response regarding data collection challenges, those without phones but with active SIM cards were supported by the study team with a spare cell phone provided at the study site, allowing them to complete peer elicitation and hash ID generation. These measures aimed to reduce exclusion bias and ensure more accurate representation of less connected or vulnerable subgroups.

**Provincial-level variations**

- While data are aggregated nationally, variations in recruitment success, network sizes, and subpopulation characteristics across provinces are not adequately explored. Analysis at the provincial level may provide insightful information and enhance local program design. Include analysis at the site and province levels to identify regional variations and inequalities in the population

Thank you for your valuable feedback. We would like to clarify that analysis at both the site and provincial levels is already included in the Results section of the manuscript. Specifically, Table 2 presents the Unadjusted and Adjusted Study Site-level Population Size Estimates of Female Sex Workers by Estimation Method, while Table 4 provides the Cross-Network Estimator's Population Size Estimates by Province, Rwanda FSW BBS 2023. These tables capture regional variations in population sizes, which are critical for informing targeted programming and resource allocation at both local and national levels.

**Discussion**

**Acknowledgment of limitations**

- While limitations (e.g., SIM card access, social desirability bias, and methodological assumptions) are noted, their potential quantitative impact on the estimates is not deeply analysed. For example, how might the lack of SIM cards influence the credibility intervals?
- Quantify the potential biases introduced by limitations like SIM card access and proportional distribution

Thank you for your comment. As noted in our earlier response to the comment on the Exclusion of FSWs without cell phones, we addressed this limitation by facilitating access to a dedicated spare mobile phone at each study site for FSWs who had SIM cards but did not own a phone. Additionally, we calculated the proportion of FSWs without access to a cell phone and used this proportion to adjust the final population size estimates accordingly. This adjustment aimed to minimize the bias introduced by their exclusion from the peer nomination process, and thus reduce the potential underestimation of the overall FSW population.

While the exact quantitative impact on the credibility intervals may be difficult to isolate, the adjustment helped mitigate possible underrepresentation. We suggested a more detailed analysis of the sensitivity of the estimates to these adjustments to be considered in future studies as part of a broader uncertainty assessment.

**PNS Assumptions and justification**

- PNS assumptions, such as randomness of connections and sufficient recruitment waves, are briefly acknowledged but not critically evaluated against observed recruitment dynamics. Were there any biases or recruitment bottlenecks?
- The methodological advantages of PNS are highlighted, but the discussion lacks a nuanced exploration of where it might still fall short compared to other methodologies, such as network-based approaches not relying on technology.
- Critically evaluate the PNS fundamental presumptions and how well they apply to the context in Rwanda.

Thank you for this important observation. We acknowledge the need to critically assess PNS assumptions in context. As described in the Data Management section of the manuscript, we conducted daily monitoring of RDS/PNS data, including close tracking of recruitment chains and peer nominations. This allowed the study team to identify and address any recruitment bottlenecks, network anomalies, or irregularities early in the process.

Through these efforts, we observed that the assumptions of sufficient recruitment waves and connectivity held reasonably well in most sites, and corrective actions were implemented where bottlenecks emerged (e.g., by adding new seeds or providing recruitment support). Furthermore, while PNS offers advantages over methods like RDS—particularly in providing population size estimates—it is not without limitations. For example, it still relies on network visibility and technological access (e.g., mobile phones), which can pose barriers in some subpopulations, as discussed in the limitations.

**Insights on hard-to-reach subgroups**

- While the study highlights its ability to capture non-venue-based FSW, the discussion does not look into specific findings about these subgroups or how their inclusion changes programmatic priorities.
- The challenges faced in reaching sexually exploited minors, as a subgroup distinct from adult FSWs, are largely unaddressed.
- Highlight specific insights gained about non-venue-based FSWs and sexually exploited minors and their implications for interventions.

Thank you for raising this point. We acknowledge the importance of examining hard-to-reach subgroups within the FSW population. In response to your comment, we have included a sentence in the revised manuscript noting that 14.3% of recruited FSWs were classified under the "Internet, phone broker, escort agency" category, as presented in Table 1. This indicates that our sampling approach successfully reached a significant proportion of non-venue-based FSWs. However, the scope of this manuscript is focused on population size estimation, and as such, it does not extend into an in-depth analysis of how subgroup representation might inform shifts in programmatic priorities. These issues warrant further research.

Regarding sexually exploited minors, the term was introduced to more accurately describe females aged 15–17 engaged in sex work, a group historically considered part of the broader FSW network in prior studies. Hence, we did not observe any particular challenge for their recruitment as compared to adults FSW.

**Implications and future directions**

- The implications for program design and policy are general and repetitive. There is limited specific guidance on how these findings should shape targeted HIV interventions or broader health programs for FSWs.
- Future research recommendations focus on smaller-area estimations but fail to suggest methodological innovations or longitudinal follow-ups to track population dynamics over time.
- Recommend broader methodological innovations and longitudinal studies for future research.

Thank you for this valuable comment. We have revised the discussion section to expand on the implications of our findings and offer more specific guidance for future programmatic and research directions. In addition to highlighting how these population size estimates support more accurate resource allocation and tailored service delivery for FSWs, we now emphasize the need for longitudinal studies to track changes in FSW population dynamics over time. This would support timely program adaptations in response to shifting patterns of sex work, mobility, and service access.

We also acknowledge the importance of methodological innovations beyond geographic disaggregation. Future research should explore the integration of real-time digital platforms, peer-assisted mobile sampling, and network-mapping technologies to improve reach and representativeness of hidden subgroups, including those operating online or in loosely connected networks. Such innovations could enhance both the robustness of size estimation and the effectiveness of targeted HIV prevention programs.
