# Supplementary material for: Estimating the size of hard to sample populations: A comprehensive study on female sex workers and sexually exploited minors in Rwanda using privatized network sampling in 2023
Source: PLoS One. 2025 Aug 21;20(8):e0329772. doi: 10.1371/journal.pone.0329772 (PMC12370040; doi:10.1371/journal.pone.0329772)
Supplement: Attachment — Submitted filename: Reviewer comments.docx [file pone.0329772.s002.docx]

**Introduction**

The research article titled “Population size estimation of female sex workers and sexually exploited minors using privatized network sampling, Rwanda, 2023” aims to estimate the population size of female sex workers (FSW) and sexually exploited minors in Rwanda using privatized network sampling (PNS), a method that uses network information collected within a bio-behavioral survey that used respondent-driven sampling (RDS). The study collected data from 2,511 FSW across 10 study sites in Rwanda from May 8th to June 24th, 2023. Three PNS estimators were used to calculate the population size: Cross-Sample, Cross-Alter, and Cross-Network. The national-level FSW population size was estimated at 98,587 (95% CI: 82,978 – 114,196), corresponding to 2.3% of the total adult female population aged 15 years and above in Rwanda. The highest proportion of FSW was found in the City of Kigali (5.3%), followed by the West Province (2.2%), and the lowest in the North Province (1.6%). The study concludes that PNS provides fundamental information for designing, planning, and implementing programs for FSW at the provincial level in Rwanda.

**Comments for improvement**

- **Title**: the title could benefit from greater clarity, ethical consideration, and sensitivity to the populations studied. A suggested title "Estimating the Size of Vulnerable Populations: A Comprehensive Study on Female Sex Workers and Sexually Exploited Minors in Rwanda using Private Network Sampling in 2023".
- **Abstract**: The abstract provides a good overview of the background, methods, results, and conclusion of the study. However, it could be improved by:
  - Providing some context and motivation for why estimating the size of FSW population is important for HIV prevention and control in Rwanda.
  - Stating the main research question or objective of the study.
  - Avoiding repetition of information, such as mentioning the study period twice or the provincial distribution of FSW twice.
- **Introduction**: The introduction provides a good background and literature review on the HIV situation among FSW in Rwanda and globally, and the challenges and methods of estimating their population size. However, it could be improved by:
  - Providing a clear statement of the research question or objective at the end of the introduction, and how it relates to the existing knowledge gap or research need.
  - Providing some hypotheses or expectations about the results of the study, based on the literature review or the formative assessment.
- **Methods**: The methods section describes the study design, procedures, sample size, data management, and statistical analysis in detail and with clarity. However, it could be improved by:
  - Providing more information about the ethical considerations, such as how the informed consent was obtained, how the confidentiality and anonymity of the participants were ensured, and how the compensation was determined and delivered.
  - Providing more information about the formative assessment, such as the number and characteristics of the FSW who participated in the group meeting and the FGD, the main findings or themes that emerged from the FA, and how they informed the design and implementation of the PNS.
  - Providing more information about the statistical analysis, such as the assumptions and limitations of the PNS estimators, and the sensitivity analysis or robustness checks performed
- **Results**:
  - Provide more interpretation and explanation of the results, such as the possible reasons or factors for the observed patterns, trends, or differences in the data, and the implications or significance of the results for the research question or objective.
- **Discussion**: The discussion section summarizes and discusses the main findings and implications of the study and compares and contrasts them with the existing literature. However, it could be improved by:
  - Providing more contextual and practical implications of the results, such as the policy and program recommendations for improving the access and quality of HIV prevention and treatment services for FSW in Rwanda, and the potential impact or benefit of the study for the HIV epidemic control and public health outcomes in the country.
  - Providing more suggestions for future research, such as the limitations or gaps that need to be addressed, the questions or hypotheses that need to be tested, the methods or data that need to be improved or developed, the collaborations or partnerships that need to be established or strengthened, and the assumptions or uncertainties of the PNS method, the representativeness or quality of the data, or the generalizability or applicability of the findings.
- **Conclusion**: The conclusion section provides a brief and concise summary of the main findings, implications, and recommendations of the study. However, it could be improved by:
  - Emphasizing the key messages or takeaways of the study and provide some directions or priorities for future research, such as the areas that need further investigation, the methods or data that need refinement or innovation, or the stakeholders or partners that need engagement or support.
- **References**:
  - Check the accuracy and completeness of the citation information, such as the authors, titles, journals, volumes, issues, pages, years, DOIs.
  - Check the consistency and correctness of the citation format, such as the punctuation, capitalization, italicization, or abbreviation of the elements of the citations.
